# Smart Semiconductors: An Overview of AZnOS (A= Ca, Sr, Ba) Based Phosphors, Multi‐Stimuli Responsive Luminescence, and Potential Application Prospects

**DOI:** 10.1002/advs.202513923

**Published:** 2025-11-12

**Authors:** Yanze Wang, Xianhui Zhang, Biyun Ren, Jingnan Zhang, Qi'an Zhang, Ziyi Fang, Mingzhi Wu, Leipeng Li, Yanmin Yang, Yixi Zhuang, Gongxun Bai, Jiulin Gan, Dengfeng Peng

**Affiliations:** ^1^ Key Laboratory of Optoelectronic Devices and Systems of Ministry of Education and Guangdong Province College of Physics and Optoelectronic Engineering Shenzhen University Shenzhen 518060 China; ^2^ College of Physics Science and Technology Hebei University Baoding 071002 China; ^3^ College of Materials and Fujian Provincial Key Laboratory of Materials Genome Xiamen University Xiamen 361005 China; ^4^ College of Optical and Electronic Technology China Jiliang University Hangzhou 310018 China; ^5^ State Key Laboratory of Luminescent Material and Devices and Guangdong Provincial Key Laboratory of Fibre Laser Materials and Applied Techniques Guangdong Engineering Technology Research and Development Center of Special Optical Fiber Materials and Devices South China University of Technology Guangzhou 510641 China; ^6^ Shenzhen Key Laboratory of Intelligent Optical Measurement and Detection Shenzhen University Shenzhen 518060 China; ^7^ State Key Laboratory of Radio Frequency Heterogeneous Integration Shenzhen University Shenzhen 518060 China

**Keywords:** AZnOS (A= Ca, Sr, Ba), multimode luminescence, opto‐electro‐mechanical response, smart semiconductors

## Abstract

Inorganic luminescent semiconductor materials doped with luminescent ions are characterized by diverse luminescence modes, high light conversion efficiency, and tunable emission wavelengths, which have been widely developed and applied in the fields of lighting, display, anti‐counterfeiting, sensing, and biomedicine. The development of new and efficient host materials with full spectral coverage has been a long‐term challenge in this field. In recent years, alkaline‐earth metal zinc oxysulfides (AZnOS, A = Ca, Sr, Ba) have become an important candidate in the field of inorganic luminescence with their excellent optical properties. The “A^2+^” and “Zn^2+^” sites in the crystal lattice provide abundant substitution sites for the doping of different luminescent ions, thus exhibiting a wide range of optical properties and tunable spectra from the visible to the near‐infrared. This review will comprehensively discuss the significant advances in the synthesis methods, optical properties of doped ions, and potential applications of AZnOS materials in recent years, especially discussing in detail the effects of rare‐earth ions doping on their luminescent properties and the corresponding tuning strategies. Additionally, this review analyzes the future direction of AZnOS materials research and the challenges of converting the research results into practical applications. It aims to provide insights for the development of inorganic luminescent semiconductor materials and advance the realization of their potential applications.

## Graphical abstract

This paper reviews the research progress of CaZnOS, SrZnOS, and BaZnOS‐based semiconductor phosphors in recent years, including their crystal structures, synthesis methods, and luminescent properties, as well as summarizes their potential applications. Finally, the challenges and opportunities for the research of these phosphors are also summarized and outlined.

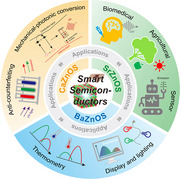

## 1 Introduction

The evolution of research on inorganic luminescent materials can be divided into four historical stages, each marked by a key scientific breakthrough. Early practice can be traced back to prehistoric mankind's simple knowledge of natural phosphorescent minerals, but systematic scientific exploration in this field began in the 19th century with the breakthrough of ion‐doping technology.^[^ ^1^, ^2^, ^3^ ^]^ In 1852, the formulation of Stokes' law laid the cornerstone of quantum theory's understanding of the mechanism of energy level transitions in luminescent materials, and it became the fundamental law of photoluminescence (PL).^[^ ^4^, ^5^ ^]^ In the 20th century, the development of relativity and quantum mechanics provided new impetus to the physics of luminescence. In 1905, Einstein explained the physical nature of Stokes' law through the quantum hypothesis of light, revealing the quantization of energy in the interaction between light and matter, and in 1913, Bohr constructed a quantum model of the atomic structure to further elucidate that the transition of electrons between the orbitals in the fixed state is the fundamental reason for the characteristic emission spectrum, which provided the core analytical framework for the subsequent design of the energy levels of doped ions.^[^ ^6^, ^7^ ^]^ The third leap in the development of the discipline occurred in the middle of the 20th century, when Maiman developed the first ruby laser in 1960, marking a breakthrough in the use of inorganic light‐emitting materials in laser technology.^[^ ^1^, ^8^, ^9^ ^]^ This event not only gave birth to a new paradigm in optoelectronics but also promoted the transformation of luminescent materials research from basic spectroscopy to functional device applications. Current research focuses on the precise modulation of material properties.^[^ ^10^ ^]^ Through the synergistic regulation of the *f*–*f* transition of rare‐earth ions (RE) and the *d*–*d* electronic states of transition metal ions (TM), researchers have realized the continuous tunable luminescence from the ultraviolet (UV) to the near‐infrared (NIR) wavelength band.^[^ ^11^, ^12^, ^13^ ^]^ This performance revolution has directly driven the industrialization of phosphor‐converted light‐emitting diodes (*pc*‐LEDs) lighting technology, which has made the optical efficacy of current commercial LED devices much higher than traditional light sources, and has made outstanding contributions to energy conservation and emission reduction.^[^ ^14^ ^,^ ^15^ ^]^

Doping is currently the most flexible technical means to modulate the luminescent properties of luminescent semiconductor materials, and by selecting suitable dopant ions, the continuously tunable characteristics of luminescent wavelengths from the UV to NIR wavelength bands can be realized.^[^ ^12^, ^16^ ^]^ With the development of PL, mechanoluminescence (ML), electroluminescence (EL), and other multimodal forms of luminescence, how to build a host material system compatible with different luminescence mechanisms has become a core scientific issue in this field.^[^ ^17^, ^18^ ^]^ In the study of multifunctional luminescent carriers, ZnS material has received extensive attention for its unique PL‐ML‐EL trimodal luminescence properties.^[^ ^19^, ^20^, ^21^, ^22^ ^]^ It is particularly noteworthy that the host exhibits excellent ML reversible properties during both elastic and plastic deformation, which has high application potential in stress sensing, information storage, and other fields.^[^ ^23^, ^24^ ^]^ However, existing studies reveal that the crystal structure contains only a single Zn^2+^ lattice site, leading to a severely limited selection of dopant ions, allowing only TM with similar radii to Zn^2+^, such as Cu^+^ and Mn^2+^, to enter the lattice.^[^ ^25^ ^]^ This structural property essentially restricts the dimensionality of luminescence wavelength tuning, which makes it difficult to meet the application requirements of multi‐band tunable luminescence. In order to break through the limitations above, current research focuses on the development of novel host crystal structures, especially material systems with multiple types of cation lattice sites.^[^ ^26^, ^27^ ^]^ These structures can achieve differential doping of luminescent ions through a selective occupancy mechanism, thereby constructing multiple luminescent centers. Through the synergistic design of lattice engineering and energy level regulation,^[^ ^28^, ^29^, ^30^ ^]^ it is expected to develop a new generation of luminescent materials with broad spectral response, high color purity, and multimode excitation characteristics, which will provide a material basis for the development of intelligent lighting, multidimensional anti‐counterfeiting, and advanced display technologies.

**Figure 1:**
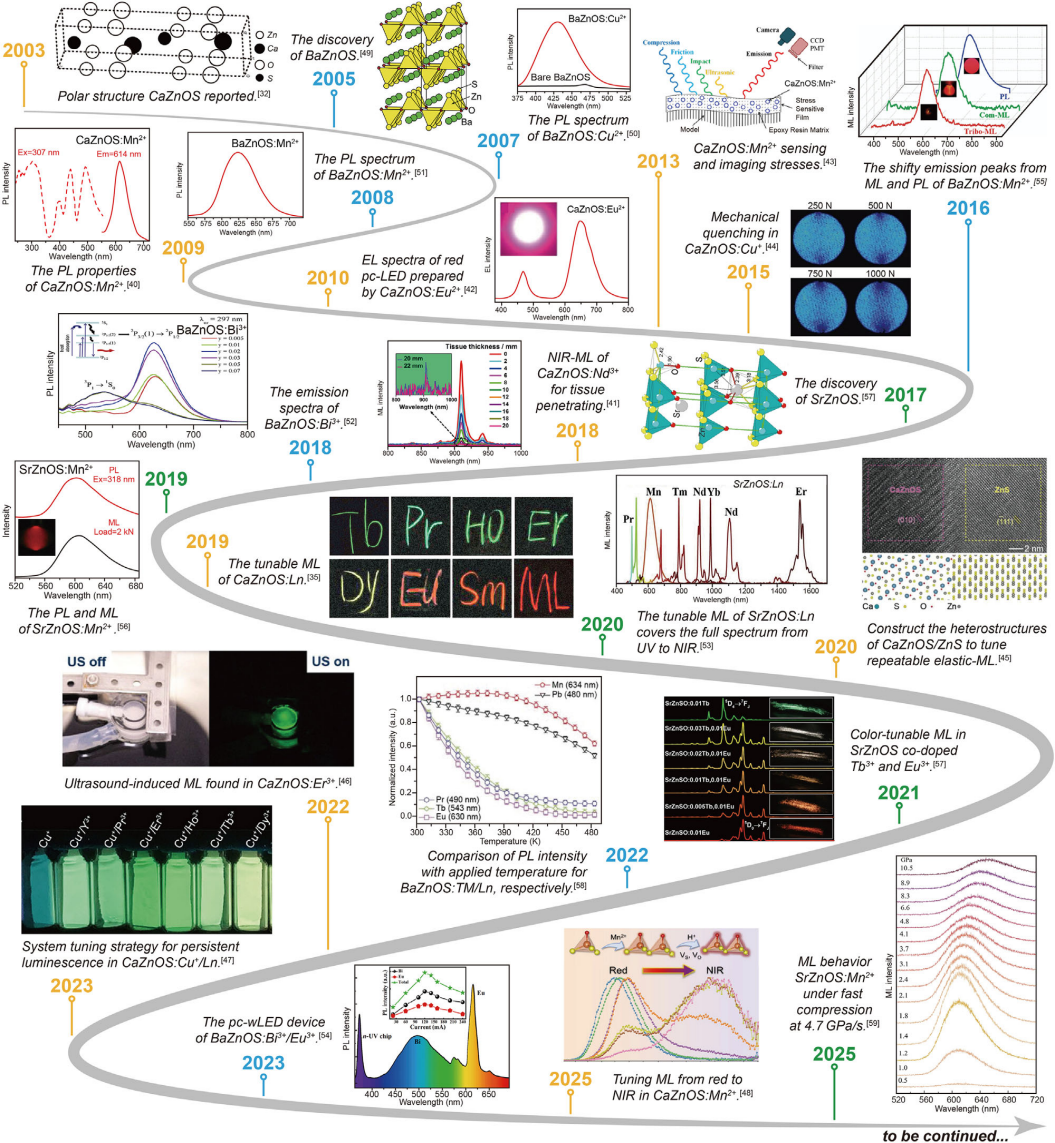
Timeline of representative research on AZnOS‐based luminescent materials. Orange timelines: representative advances in the development and application of CaZnOS. Reproduced with permission.^[^ ^32^ ^]^ Copyright 2003, Pleiades; Reproduced with permission.^[^ ^40^, ^41^ ^]^ Copyright 2009, 2018, American Chemical Society; Reproduced with permission.^[^ ^42^, ^43^ ^]^ Copyright 2010, 2013, Optical Society; Reproduced with permission.^[^ ^44^ ^]^ Copyright 2015, Nature; Reproduced with permission.^[^ ^35^, ^45^, ^46^, ^47^, ^48^ ^]^ Copyright 2019, 2020, 2022, 2023, 2025, Wiley‐VCH. Green and blue timelines: the discovery of SrZnOS and BaZnOS, as well as the development of their luminescent properties, respectively. Reproduced with permission.^[^ ^49^ ^]^ Copyright 2005, American Chemical Society; Reproduced with permission.^[^ ^50^, ^51^, ^52^, ^53^, ^54^ ^]^ Copyright 2007, 2008, 2018, 2020, 2023, Elsevier; Reproduced with permission.^[^ ^55^, ^56^ ^]^ Copyright 2016, 2019, The Royal Society of Chemistry; Reproduced with permission.^[^ ^57^, ^58^ ^]^ Copyright 2021, 2022, Wiley‐VCH; Reproduced with permission.^[^ ^59^ ^]^ Copyright 2025, Nature.

Quaternary alkaline‐earth metal oxysulfides with the molecular formula AZnOS (A = Ca, Sr, Ba) can be regarded as an important derivation system of ZnS, and their crystal structure provides a unique coordination environment for Zn^2+^.^[^ ^31^ ^]^ The additional “A^2+^” sites in these materials exhibit excellent inclusivity to dopants, such as RE, providing a structural basis for modulating luminescence performance. Among them, CaZnOS‐based materials have become a model for the study of this system due to their unique physicochemical properties (**Figure** **1**, orange timelines). The synthesis of CaZnOS started in 2003, when Petrova et al. observed the compound for the first time during the reduction procedure of the ZnS recycling process.^[^ ^32^ ^]^ In 2007, Clark's group succeeded in the preparation of the high‐purity phase CaZnOS through a thermodynamically controlled synthesis strategy, and established a controlled synthesis methodology to achieve the high‐purity phase CaZnOS.^[^ ^33^ ^]^ In terms of optical property studies, in 2015, Zhang et al. systematically investigated the Mn^2+^ concentration‐dependent PL properties in the CaZnOS: Mn^2+^, revealing the phenomenon of successive red‐shifts of the emission peak position with the elevation of the doping concentration.^[^ ^34^ ^]^ In 2019, Wang and colleagues further reported that CaZnOS's efficient accommodation capacity for lanthanide ions (Ln), and realized a tunable ML covering the visible light (VIS) to NIR band by doping with Ln such as Tb^3+^, Sm^3+^, and Nd^3+^.^[^ ^35^ ^]^ After nearly two decades of continuous exploration, CaZnOS‐based luminescent materials have formed a complete research system of material design‐property tuning‐application exploration.^[^ ^36^, ^37^, ^38^, ^39^ ^]^ These advances provide an important platform for the development of new smart sensing materials and broad‐spectrum light sources.

In addition to the CaZnOS, important progress has been made in the study of SrZnOS and BaZnOS. Synthesis studies for SrZnOS showed significant challenges in the preparation of single‐phase materials, despite its isostructural crystal structure with CaZnOS. It was not until 2017 that Valldor's group obtained pure‐phase SrZnOS for the first time by optimizing the synthesis process using Zn powder, SrO, and sulfur powder as raw materials and a high‐temperature solid‐ state reaction at 1050 °C for 10 h (Figure 1, green timelines).^[^ ^60^ ^]^ It was shown that SrZnOS could be synthesized in the calcination temperature interval of 1000–1080 °C, but this temperature range was easily accompanied by the generation of the by‐product SrZn_2_OS_2_, which became a key factor restricting the acquisition of single‐phase SrZnOS crystals.^[^ ^61^, ^62^ ^]^ In 2020, Xie's research group, by systematically carrying out Ln/TM doping studies, revealed that SrZnOS had the capability to be used from the VIS to NIR wavelengths, and its emission characteristics are closely related to the dopant energy level structure.^[^ ^53^ ^]^ Especially noteworthy is that Yang's group in 2025 observed anomalous ML kinetic behavior in SrZnOS: Mn^2+^, and found that the heating rate and excitation temperature have a non‐monotonous modulation effect on the ML intensity.^[^ ^59^ ^]^ Compared to the research progress of SrZnOS, the synthesis and study of BaZnOS show different features. In 2005, Clarke's team stumbled upon BaZnOS while exploring novel oxysulfide systems (Figure 1, blue timelines), laying a material foundation for subsequent studies.^[^ ^49^ ^]^ In 2018, Peng and colleagues achieved a major breakthrough in the synthesis and study of BaZnOS: Mn^2+^. They observed for the first time a significant elastic deformation‐induced ML effect, with a positive correlation between luminescence intensity and strain magnitude.^[^ ^55^ ^]^ Wang et al. in 2022 further show that both Ba^2+^ and Zn^2+^ double lattice sites of BaZnOS are inclusive of dopant ions, enabling selective occupancy doping of Ln/TM.^[^ ^58^ ^]^ It is noteworthy that spectral analysis shows a significant correlation between the luminescence intensity of the doped ions and the test temperature. However, up to now, no active luminescence of other Ln has been realized in BaZnOS except for the BaZnOS: Mn^2+^, which may be related to the energy level concatenation effect due to the high symmetry of the BaZnOS crystal structure,^[^ ^55^, ^63^ ^]^ and the mechanistic study of this phenomenon is being carried out in depth.

As an emerging quaternary compound system in the last decade, AZnOS provides a structural basis for constructing multiple luminescent centers through its unique dual‐cation lattice sites and synergistic doping of different luminescent ions achieved by design. Based on crystal field modulation and energy level engineering principles, such materials are expected to achieve breakthrough progress in the field of smart optoelectronic devices through optimized precision doping strategies. This review systematically compiles the latest research progress of AZnOS‐based phosphors, covering the dimensions of crystal structure analysis, luminescence mechanism research, synthesis process optimization, and multi‐scenario application exploration. In particular, the tuning laws of different doping strategies on optical properties are systematically organized, including the energy transfer mechanism and spectral tuning methods in the co‐doped system. Among them, the CaZnOS becomes the focus of research due to its excellent ML properties, and its fast response to external mechanical stimuli and reproducibility are highlighted in this review. In terms of application prospects, this review systematically summarizes the potential application paths of AZnOS‐based phosphors in the fields of solid‐state lighting, sensing, anti‐counterfeiting, and biomedical. Therefore, by integrating the research progress of the whole chain of “structure–property–application,” this review expects to provide a systematic knowledge framework for researchers in the field of inorganic luminescent materials, as well as a theoretical guidance and technical reference for the development of the new generation smart phosphors.

## 2 The Crystal and Optoelectronic Structures of AZnOS

The crystal and electronic structures exert a decisive influence on the luminescent properties of phosphors. By studying these two structures, we can gain deeper insights into crucial factors such as energy transfer, excitation–emission mechanisms, and thermal stability during the luminescence process.^[^ ^64^, ^65^ ^]^ This understanding enables the optimization of phosphor design and application, enhancing their performance in fields such as display technology, lighting, lasers, sensors, and more.

**Figure 2:**
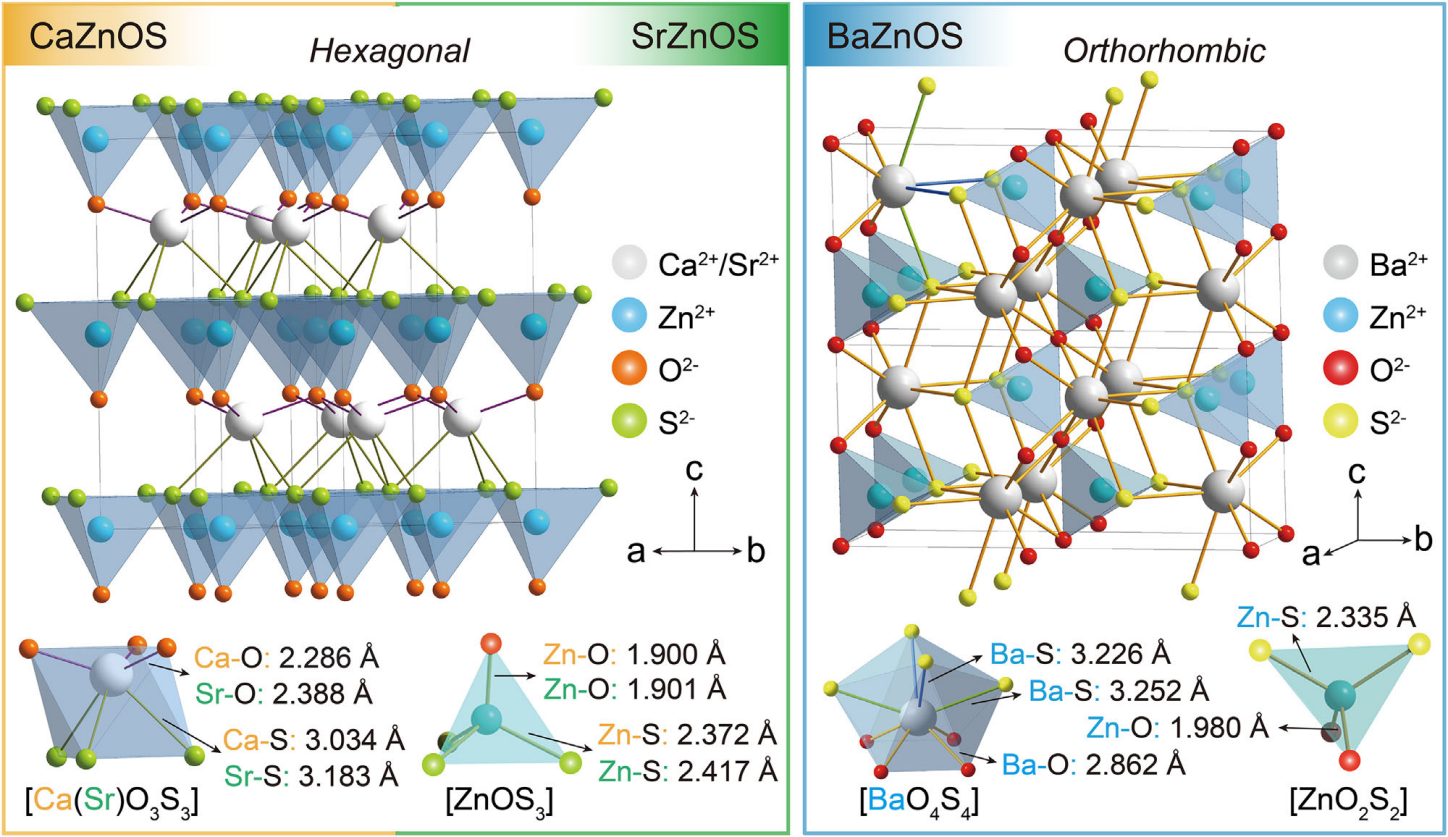
The crystal structure of AZnOS (top), along with the coordination environments and bond lengths of different “A^2+^” and Zn^2+^ (bottom inset).

Hence, this review first introduces the crystal structure of AZnOS and its related physical properties. As illustrated in **Figure** **2**, the AZnOS lattice comprises close‐packed sulfur and oxygen layers arranged in a hexagonal close‐packed stacking sequence.^[^ ^25^, ^53^ ^]^ CaZnOS and SrZnOS share the same non‐centrosymmetric hexagonal structure, while BaZnOS adopts a high‐symmetry orthorhombic structure. Within these structures, Ca^2+^/Sr^2+^ occupy octahedral sites, Ba^2+^ occupies decagonal sites, and Zn^2+^ occupies both tetrahedral sites. The 3D framework comprises infinite layers of [Ca(Sr)O_3_S_3_] octahedra, [BaO_4_S_4_] dodecahedra, and vertex‐connected [ZnOS_3_] or [ZnO_2_S_2_] tetrahedra. These units interconnect via S─S edges and O vertices. A notable structural feature lies in the *a*/*b*‐axis layers, where polar characteristics arise from the shared [ZnOS_3_] rather than [ZnO_2_S_2_] layers.^[^ ^42^, ^60^, ^66^ ^]^ These polar layers of these 4‐coordinate geometries are interspersed with [Ca(Sr)O_3_S_3_] layers, where Ca^2+^/Sr^2+^ sites exhibit 6‐coordinate geometries. In the [BaO_4_S_4_] layers, Ba^2+^ sites adopt 8‐coordinate geometry. The alternating stacking of octahedral/decahedral and tetrahedral layers confers structural polarity, influencing the material's optical and electronic properties. Specific details of each crystal are described below.

CaZnOS crystallizes in a non‐centrosymmetric hexagonal system with the space group of *P6_3_mc*.^[^ ^32^ ^]^ The lattice parameters are a = b = 3.75726(3) Å and c = 11.4013(1) Å. The structure comprises two puckered hexagonal layers (CaO and ZnS), where Zn^2+^ is tetrahedrally coordinated by three S atoms (from ZnS layers) and one O atom (from adjacent CaO layers), forming distorted octahedral environments.^[^ ^34^, ^67^ ^]^ Ca^2+^ occupies distorted octahedral interstices between these layers. Competition between Zn^2+^ and Ca^2+^ for O^2−^ drives the formation of polar [ZnOS_3_], enhancing luminescence across multiple modes.

SrZnOS, isostructural to CaZnOS, also adopts a polar *P6_3_mc* space group but exhibits a larger unit cell (a = b = 3.90442(6) Å, c = 11.6192(2) Å) due to the larger Sr^2+^ ionic radius.^[^ ^53^ ^]^ Its structure consists of S‐vertex‐sharing [ZnOS_3_] forming triangular layers, separated by Sr^2+^ in distorted octahedra. Both Sr^2+^ and Zn^2+^ sites exhibit strong polarity due to non‐centrosymmetric S^2−^/O^2−^ distributions and ionic radius differences (*r*_O_^2−^ = 1.26 Å, *r*_S_^2−^ = 1.70 Å), forcing cation displacements toward O‐rich regions.

In contrast, BaZnOS crystallizes in the orthorhombic space group *Cmcm* (a = 3.9616(2) Å, b = 12.8541(7) Å, c = 6.1175(4) Å). Zn^2+^ is tetrahedrally coordinated by two O and two S atoms, forming [ZnO_2_S_2_] that share vertices to create chains along the *a*‐ and *c*‐axes.^[^ ^55^ ^]^ Puckered Zn─O─S layers are separated by Ba^2+^ [4×O at 2.8616(2) Å, 2×S at 3.2263(8) Å, and 2×S at 3.2523(4) Å], arranged in a distorted bisymmetric triangular prism.

**Figure 3:**
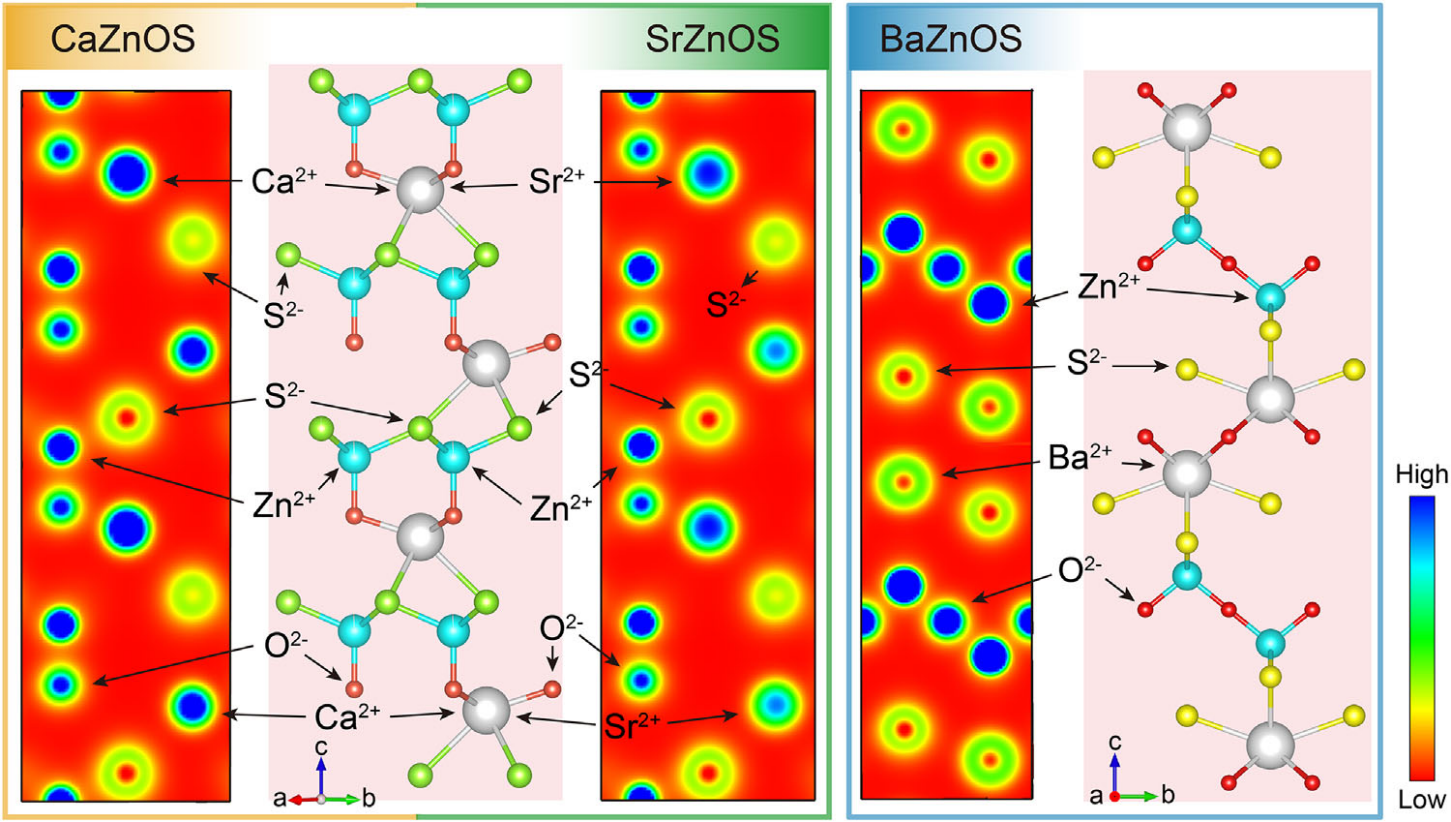
Charge density corresponding to each element in AZnOS crystals.

The optoelectronic properties of AZnOS were subsequently discussed, with a focus on its band structures, electronic transitions, and their potential impact on optical characteristics. Understanding these aspects is critical for predicting its performance in optoelectronic mechanical systems. Its optoelectronic behavior is further influenced by non‐centrosymmetric crystal structures and localized units, with specific structural parameters playing key roles.^[^ ^68^ ^]^ The gradual increase in the radius of the “A^2+^” leads to a consequent expansion of the lattice parameters of AZnOS, resulting in distinct physical properties. For example, as shown in **Figure** **3**, the charge densities of Ca^2+^, Sr^2+^, and Zn^2+^ cations are approximately equivalent in CaZnOS and SrZnOS. In BaZnOS, however, the weakened metallicity of Ba^2+^ reduces its electron density, thereby weakening semiconductor conductivity.^[^ ^69^ ^]^ Additionally, the bandgap decreases with increasing “A^2+^” ionic radius, shifting optical absorption toward lower energies (red‐shift).

The bandgap of CaZnOS (≈3.71 eV) is wider than SrZnOS (≈3.10 eV), indicating that SrZnOS is more covalently bonded.^[^ ^66^, ^70^ ^]^ BaZnOS has a direct band gap of ≈3.91 eV, which is the largest in AZnOS and even wider than ZnS (≈3.67 eV).^[^ ^58^ ^]^ Density of electronic states (DOS) calculations show that the valence band (VB) is formed mainly by the hybridization of O 2*p* and S 3*p*, and the conduction band (CB) is formed mainly by Zn 4*s* states.^[^ ^71^, ^72^ ^]^ A comprehensive understanding of the optical and electronic structure of AZnOS is essential to advance its applications.

## 3 Luminescent Properties of Doped Ions

**Figure 4:**
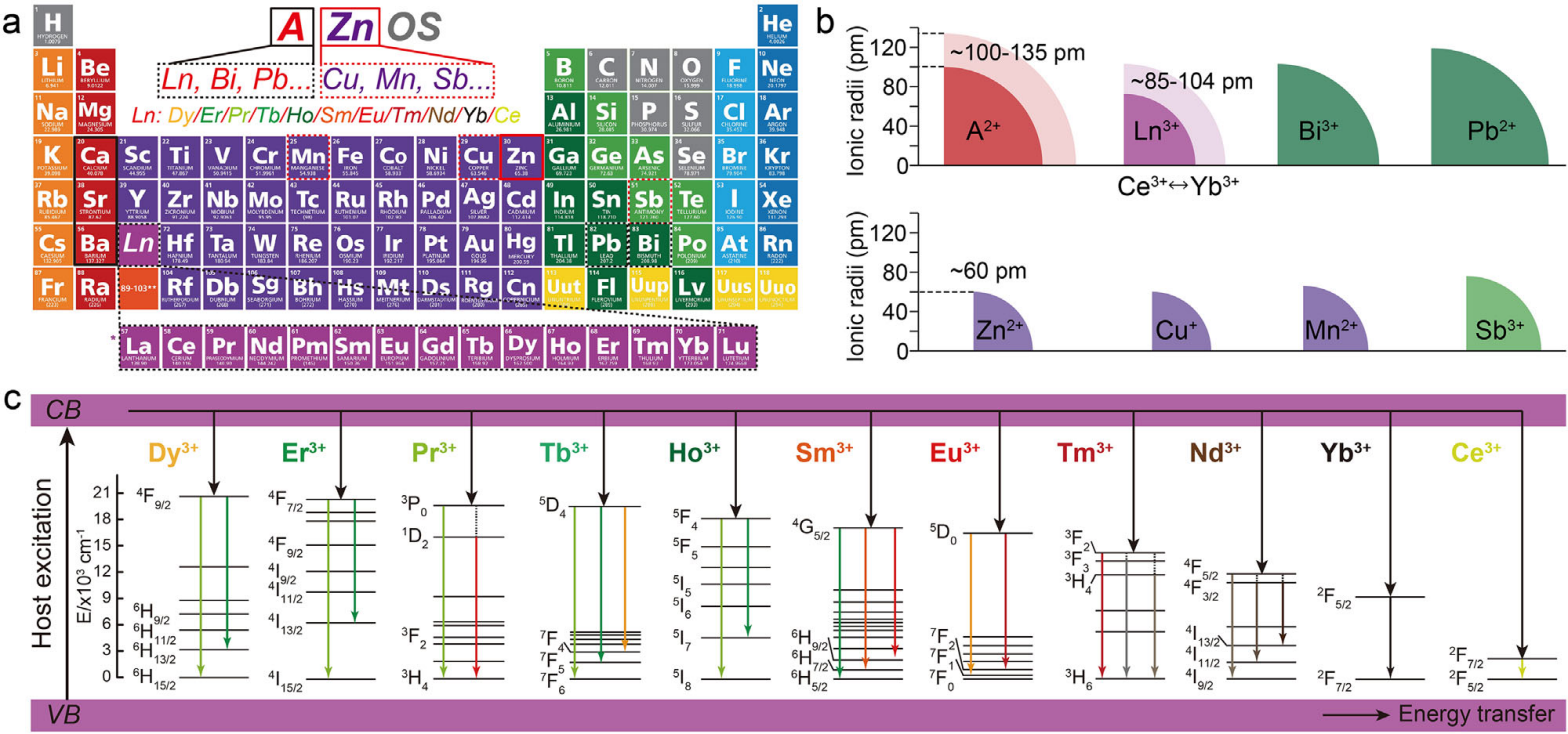
a) The position of each element in AZnOS in the Periodic Table and the doping ions that can be accommodated at the “A^2+^” and Zn^2+^ sites. b) Comparison of ionic radii (according to Shannon's formula) between typical luminescent activators and cations in AZnOS. Reproduced with permission.^[^ ^47^ ^]^ Copyright 2023, Wiley‐VCH. c) A schematic energy level diagram for the energy transfer process of Ln^3+^ (i.e., Dy^3+^, Er^3+^, Pr^3+^, Tb^3+^, Ho^3+^, Sm^3+^, Eu^3+^, Tm^3+^, Nd^3+^, Yb^3+^, and Ce^3+^)‐doped AZnOS.

AZnOS have been extensively investigated as efficient host materials for converting external stimuli (e.g., radiation, mechanical stress) into light emission.^[^ ^72^ ^]^ This system not only exhibits a wide bandgap but also provides versatile coordination environments through its unique bication sites. Such structural versatility constitutes a critical advantage, enabling AZnOS to outperform many conventional inorganic phosphors. During doping processes, ion substitution occurs via replacement of host lattice sites by dopant ions, necessitating favorable ionic radius compatibility between dopants and host cations.^[^ ^73^, ^74^, ^75^ ^]^ As shown in **Figures** **4**a, the ionic radii of the Group IIA cations (Ca^2+^: ≈99 pm, Sr^2+^: ≈113 pm, Ba^2+^: ≈135 pm) increase sequentially. Consequently, Ln^3+^ (≈85.8─103.4 pm), Bi^3+^ (≈108 pm), and Pb^2+^ (≈120 pm) with matching radii can theoretically occupy these lattice positions. Similarly, Group IIB Zn^2+^ sites may be substituted by Cu^2+^ (≈72 pm), Mn^2+^ (≈80 pm), and Sb^3+^ (≈92 pm) (Figure 4b), demonstrating the AZnOS's capacity to accommodate dopants with ionic radii spanning ≈60–140 pm.^[^ ^47^ ^]^ This broad radius compatibility facilitates the creation of highly tunable luminescent platforms. Notably, Ln possess unique 4*f* electron configurations (Figure 4c) that enable rich energy level transitions in their outer shells upon external excitation.^[^ ^65^, ^76^ ^]^ This results in broadband emission spanning the VIS to NIR spectral region. The exceptional spectral tunability of Ln^3+^‐doped AZnOS makes them ideal models for investigating dopant‐host interactions.

Building on these structural attributes, this review systematically analyzes the luminescence characteristics of various dopant categories within AZnOS, and the modulation effects of local coordination environments on dopant energy levels and photophysical processes. This comprehensive analysis aims to establish structure–property relationships that will guide the development of advanced multifunctional luminescent materials.

### 3.1 Ln‐Doped Luminescence

**Figure 5:**
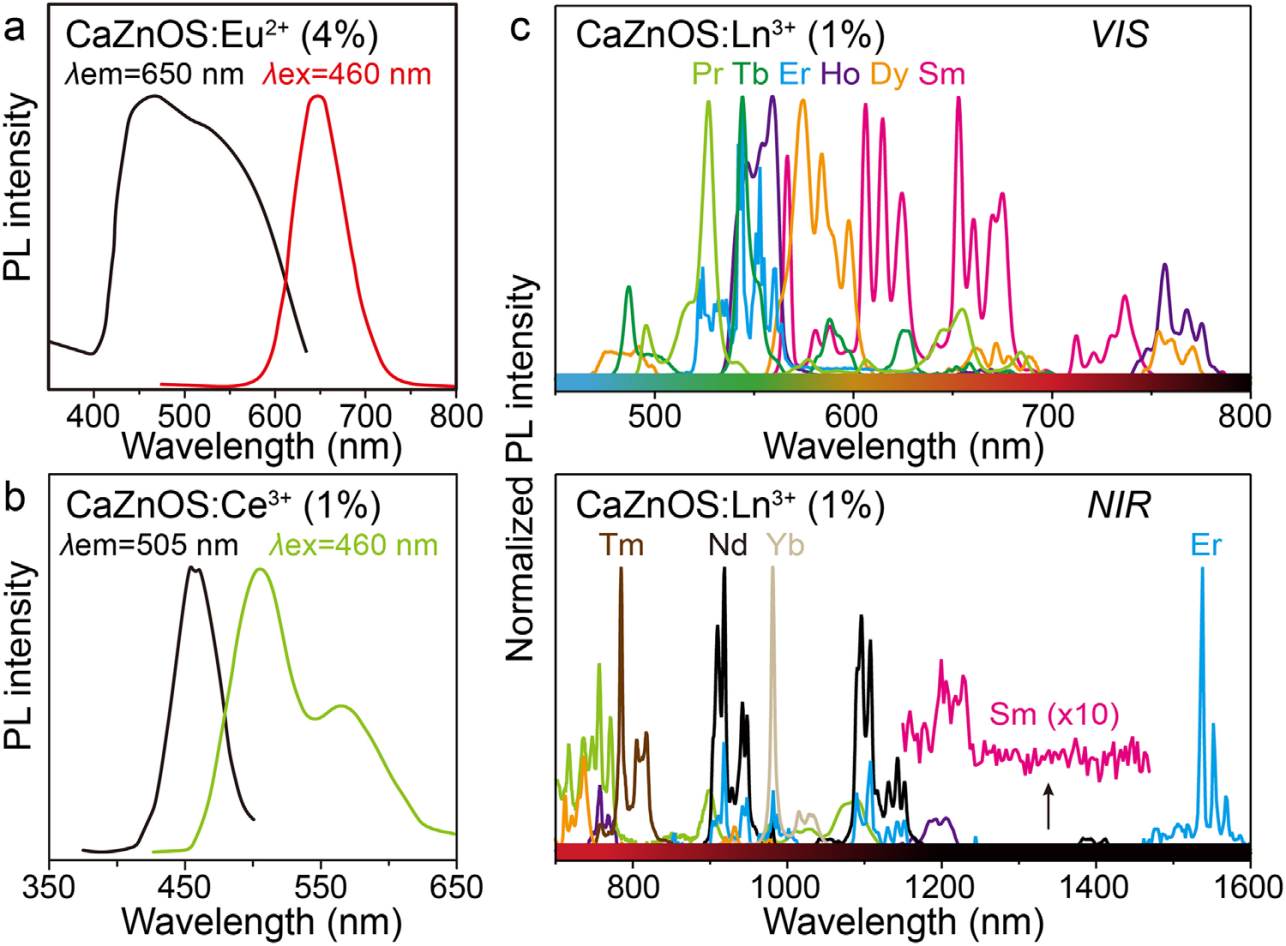
Excitation (black) and emission (color) spectra of a) CaZnOS: Eu^2+^ and b) CaZnOS: Ce^3+^, respectively. Reproduced with permission.^[^ ^42^ ^]^ Copyright 2010, Optical Society of America. Reproduced with permission.^[^ ^82^ ^]^ Copyright 2014, Elsevier. c) Emission spectra of CaZnOS: Ln^3+^ (Ln = Tb^3+^, Pr^3+^, Ho^3+^, Dy^3+^, Eu^3+^, Sm^3+^, Er^3+^, Tm^3+^, Nd^3+^, and Yb^3+^).

Early studies on AZnOS focused on the light‐emitting properties in the UV to VIS wavelength bands. However, with the development of science and technology, there is a growing demand to extend the light emission range to the NIR and even the mid‐ and far‐infrared bands, which is strategically important for biomedical imaging, optical communication technology, and other fields.^[^ ^75^, ^77^ ^]^ To meet this demand, Ln‐doped luminescent materials have become a research topic in this field. However, realizing effective doping of Ln in host materials is extremely challenging, which mainly stems from the limited compatibility between Ln and host materials, the need for precise control of key parameters, such as host/dopant combinations and dopant concentration distribution.^[^ ^78^, ^79^, ^80^, ^81^ ^]^ Most of the early studies focused on the *d*–*f* transition doping systems of Ce^3+^ and Eu^2+^ (**Figure** **5**a,b). Experiments show that the CaZnOS: Eu^2+^ and CaZnOS: Ce^3+^ exhibit broadband absorption properties in the wavelength range of 450–500 nm, and their emission spectra exhibit a broadband absorption in the range of 650 nm (Eu^2+^) and 505 nm (Ce^3+^), centered broadband emission.^[^ ^42^, ^82^ ^]^ Until 2013, Zhang et al. systematically reported for the first time the PL properties of various Ln^3+^, such as Pr^3+^, Sm^3+^, Er^3+^, Tm^3+^, doped into the CaZnOS.^[^ ^66^ ^]^ It has been observed that these ions exhibit typical *f*–*f* transition absorption and emission properties from the VIS to the NIR region in CaZnOS (Figure 5c).^[^ ^23^ ^]^ Notably, the study of Zhang et al. visualized the energy level positional relationships of the 4*f* and 5*d* energy levels of Ln relative to the top of the VB and the bottom of the CB of CaZnOS by constructing an energy level alignment diagram. This energy level structure analysis provides an important theoretical basis for understanding the PL properties and valence stability of Ln; that is, when there is a large energy gap between the ground state energy levels of divalent Ln and the CB bottom of the host material, the divalent state is more likely to exist stably during the material preparation process.^[^ ^83^ ^]^ For example, the 4*f* ground state of Eu^2+^ is closer to the top of the VB, which makes it tend to maintain a divalent stabilized state in CaZnOS; whereas the 4*f* energy levels of Ce^3+^, Sm^3+^, and Tm^3+^ are closer to the bottom of the CB, which results in the fact that it is easier for them to exist in a trivalent form (Figure 4c).^[^ ^66^ ^]^

**Figure 6:**
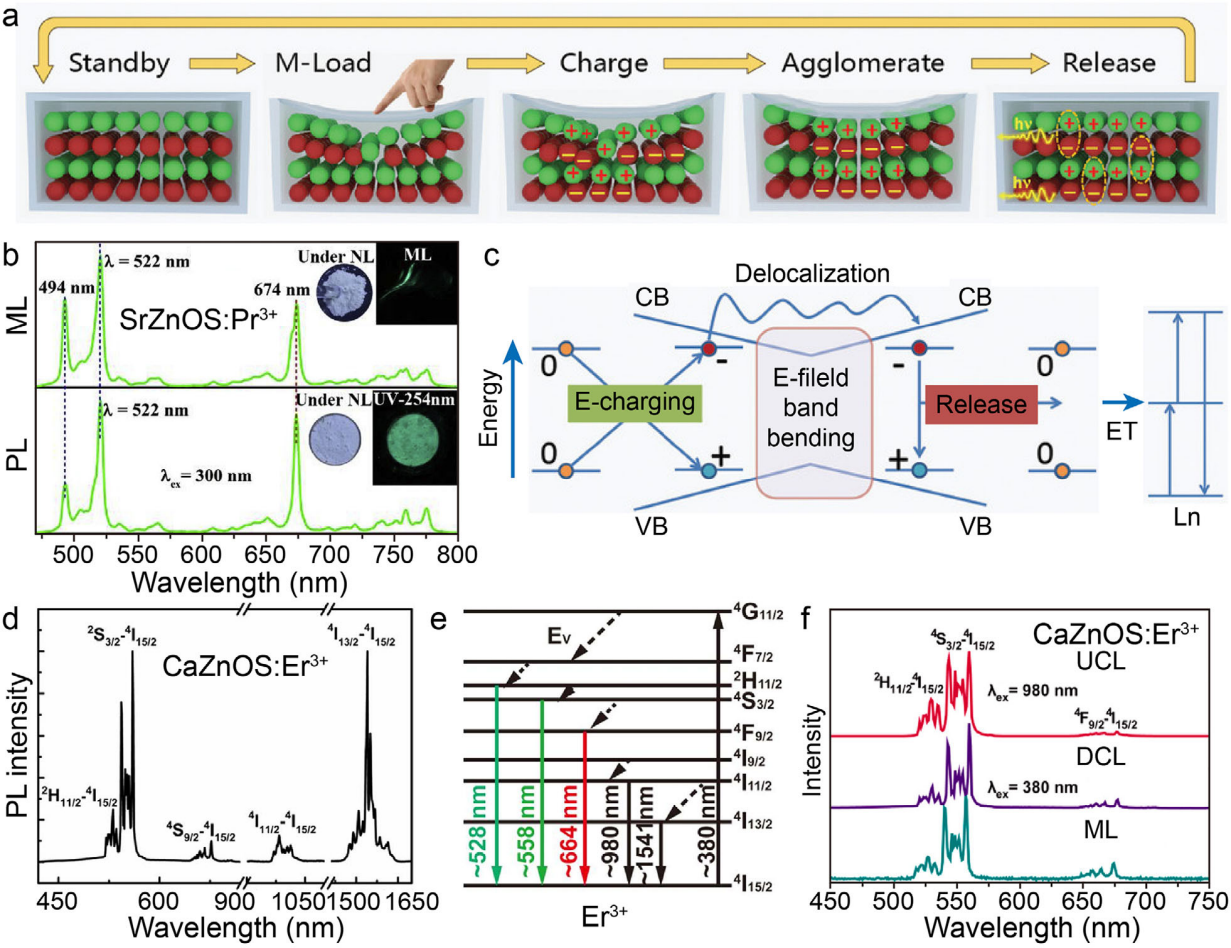
a) Proposed ML mechanism in the Ln^3+^‐doped CaZnOS crystals. Reproduced with permission.^[^ ^35^ ^]^ Copyright 2019, Wiley‐VCH. b) ML (top) and PL spectra (bottom) of SrZnOS: Pr^3+^, respectively. The insets show photos of the sample under natural light, under UV excitation, and upon rubbing by using a glass rod, respectively. Reproduced with permission.^[^ ^53^ ^]^ Copyright 2020, Elsevier. c) The overall energy diagram for an electronic transition within a neutral charge environment. E‐field is electric field; E‐Charging is E‐field induced charge‐separation; ET is energy transfer. Reproduced with permission.^[^ ^35^ ^]^ Copyright 2019, Wiley‐VCH. d) Emission spectrum of CaZnOS: Er^3+^ from VIS to NIR region and e) the energy level diagram corresponding to Er^3+^. f) UCL, DCL, and ML spectra of CaZnOS: Er^3+^, respectively. Reproduced with permission.^[^ ^85^ ^]^ Copyright 2015, American Chemical Society. All spectra were obtained at room temperature.

Both CaZnOS and SrZnOS are direct bandgap semiconductors belonging to the hexagonal crystal system. Their polar non‐centrosymmetric structures endow these materials with excellent piezoelectric properties, providing a structural foundation for efficient piezoelectric–optical coupling conversion (**Figure** **6**a).^[^ ^35^, ^84^ ^]^ When Ln are doped into the Ca(Sr)ZnOS lattice, significant PL properties arise from *f*–*f* transitions under both UV excitation and mechanical stress (Figure 6b). Taking SrZnOS: Pr^3+^ as an example, characteristic emission peaks are observed at 494, 522, 674, 759, and 775 nm (bottom of Figure 6b).^[^ ^53^ ^]^ Notably, SrZnOS: Pr^3+^ samples under mechanical stress exhibit green light that is highly consistent with the PL spectra (top of Figure 6b). The physical mechanism underlying this ML effect can be elucidated as follows (Figure 6c): mechanical stress applied to the non‐centrosymmetric Ca(Sr)ZnOS lattice generates piezoelectrically polarized charges. These charges are subsequently trapped by intrinsic lattice defects (e.g., oxygen vacancies), inducing electron–hole recombination. The energy released from this recombination process is non‐radiatively transferred to doped Ln, ultimately exciting their characteristic ML emissions.^[^ ^25^, ^35^ ^]^ Leveraging the rich energy level structure of Ln, the doped systems enable multi‐band tunable ML emission. While the emission wavelength is modulated by the choice of Ln^3+^ species (e.g., Tb^3+^/Ho^3+^/Pr^3+^ for green emission, Dy^3+^ for yellow emission, Sm^3+^ for red emission, and Tm^3+^/Nd^3+^/Yb^3+^ for NIR emission), the 4*f* electronic configuration's shielding effect ensures intrinsic spectral stability. The Er^3+^ doped sample further exemplifies unique luminescence behavior: its stepped energy level configuration enables CaZnOS: Er^3+^ to exhibit bimodal emission in both the green (^2^ *H*_11/2_/^4^ *S*_3/2_ → ^4^ *I*_15/2_) and NIR (^4^ *I*_13/2_ → ^4^ *I*_15/2_) regions (Figure 6d and e).^[^ ^85^ ^]^ Remarkably, Er^3+^ demonstrates simultaneous up‐conversion luminescence (UCL), down‐conversion luminescence (DCL), and ML emission modes within the Ca(Sr)ZnOS. Spectral comparison of a single CaZnOS: Er^3+^ sample (Figure 6f) reveals that Er^3+^ emission peaks remain highly consistent across all three excitation modes. This indicates that Ln luminescence in Ca(Sr)ZnOS is excitation‐source‐independent, with intrinsic emission wavelengths unaffected by changes in excitation modalities.^[^ ^86^, ^87^ ^]^

**Figure 7:**
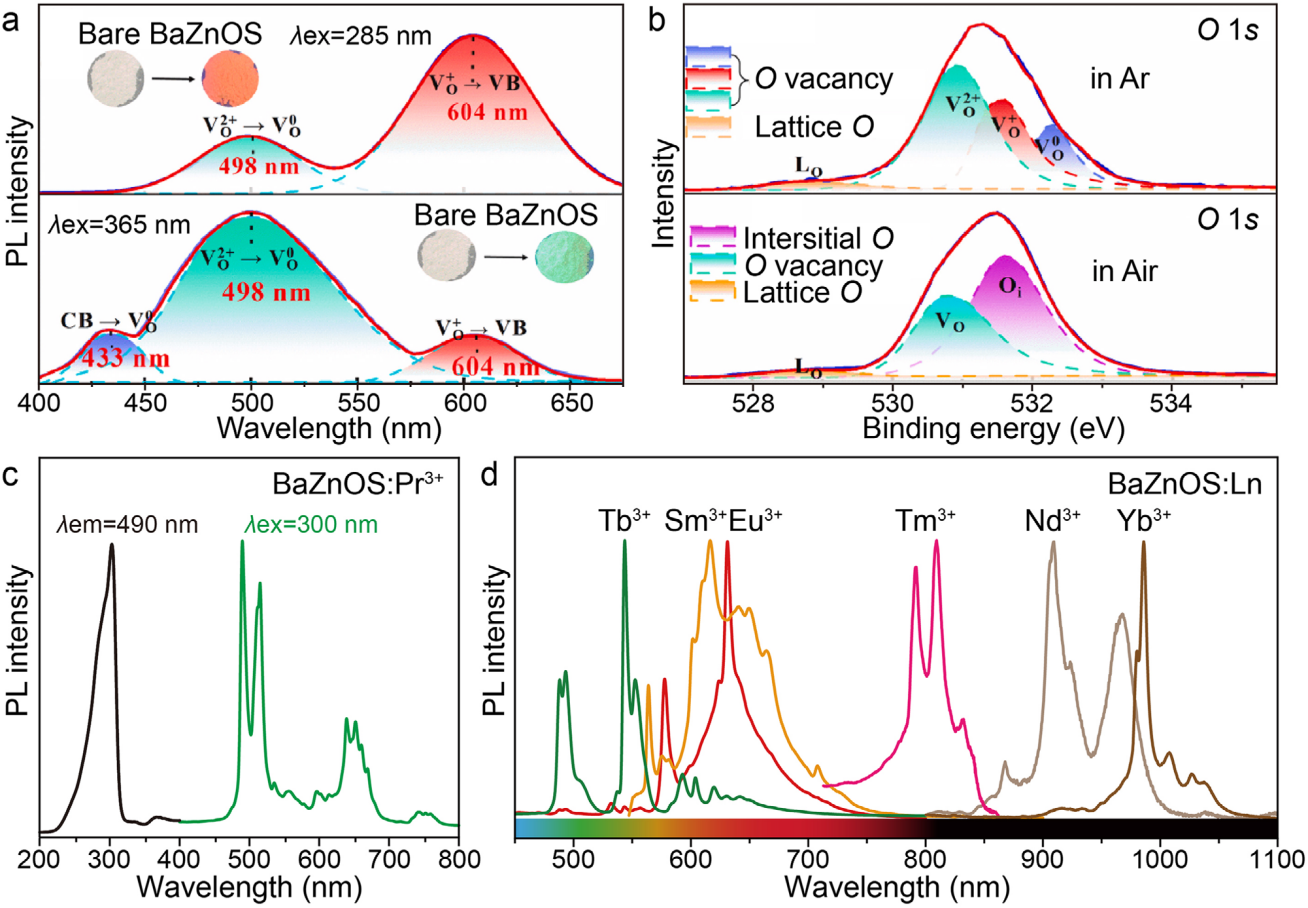
a) Multi‐colored self‐PL and optical digital images of the bare BaZnOS under *λ*ex = 285 and 365 nm, respectively. b) O 1*s* XPS spectra of the bare BaZnOS sintered under Ar and air atmosphere, respectively. Reproduced with permission.^[^ ^54^ ^]^ Copyright 2023, Elsevier. c) Excitation and emission spectra of BaZnOS: Pr^3+^ and d) emission spectra of BaZnOS doped with various Ln^3+^ (i.e., Tb^3+^, Sm^3+^, Eu^3+^, Tm^3+^, Nd^3+^, and Yb^3+^) under *λ*ex = 300 nm. Reproduced with permission.^[^ ^58^ ^]^ Copyright 2022, Wiley‐VCH. All spectra were obtained at room temperature.

Unlike Ca(Sr)ZnOS, which exhibits a non‐centrosymmetric crystal structure, BaZnOS adopts a centrosymmetric configuration, rendering its non‐polar lattice inherently unsuitable for ML manifestation upon Ln doping.^[^ ^58^, ^63^ ^]^ Notably, the ionic radius of Ba^2+^ exceeds that of Ca^2+^ and Sr^2+^ in the Group IIA cations, providing a more compatible lattice environment for Ln incorporation. However, as demonstrated in **Figure** **7**a, the synthesis of bare BaZnOS under Ar atmosphere often induces multiple lattice imperfections.^[^ ^54^ ^]^ Under 285 nm (top panel) and 365 nm (bottom panel) excitations, broad emission bands centered at 433, 498, and 604 nm were observed, indicative of defect‐related radiative recombination pathways. Further XPS study revealed that, through comparison of bare BaZnOS synthesized under Ar and ambient atmospheres via analysis (Figure 7b), these defect emissions originate from the synergistic contributions of intrinsic oxygen vacancies (*V*_O_^2+/+/0^), interstitial oxygen (*O*_i_), and lattice oxygen (*L*_O_).^[^ ^88^, ^89^ ^]^ Post Ln doping, all samples exhibit tunable emission spanning green to NIR regions when excited through the host's characteristic ≈300 nm absorption band (Figure 7c,d).^[^ ^58^ ^]^ Notably, the spectral overlap between Ln emission peaks and defect‐induced broad bands (especially found in Eu^3+^ and Sm^3+^) suggests suboptimal energy transfer efficiency from the host lattice to dopant ions when samples are synthesized under inert conditions.^[^ ^50^ ^]^

### 3.2 TM and Other Luminescent Ions Doped Luminescence

The Zn^2+^ sites in the AZnOS lattice provide an ideal coordination environment for the doping of TM, among which Mn^2+^ and Cu^+^ become the key emission centers for realizing spectral modulation of the AZnOS due to their unique *d*‐orbital electron configuration and strong coupling with the crystal field.^[^ ^90^ ^]^ Specifically, the 3*d* ^5^ electronic configuration of Mn^2+^ induces a significant crystal field splitting effect within the tetrahedral ligand field, whereas the 3*d* ^10^ electronic configuration of Cu^+^ forms a unique charge transfer state through *p*–*d* hybridization with anionic ligands.^[^ ^91^ ^]^ The synergistic interaction between these two dopants provides a multidimensional emission modulation pathway for the AZnOS.^[^ ^56^, ^71^, ^92^ ^]^ Furthermore, significant progress has been made in studies involving the doping of non‐traditional luminescent center ions (such as Bi^3+^, Sb^3+^, and Pb^2+^) into AZnOS. Experiments have confirmed that these ions can generate characteristic emission through strong interactions between their 6*s* ^2^ lone pair electrons and the lattice.

**Figure 8:**
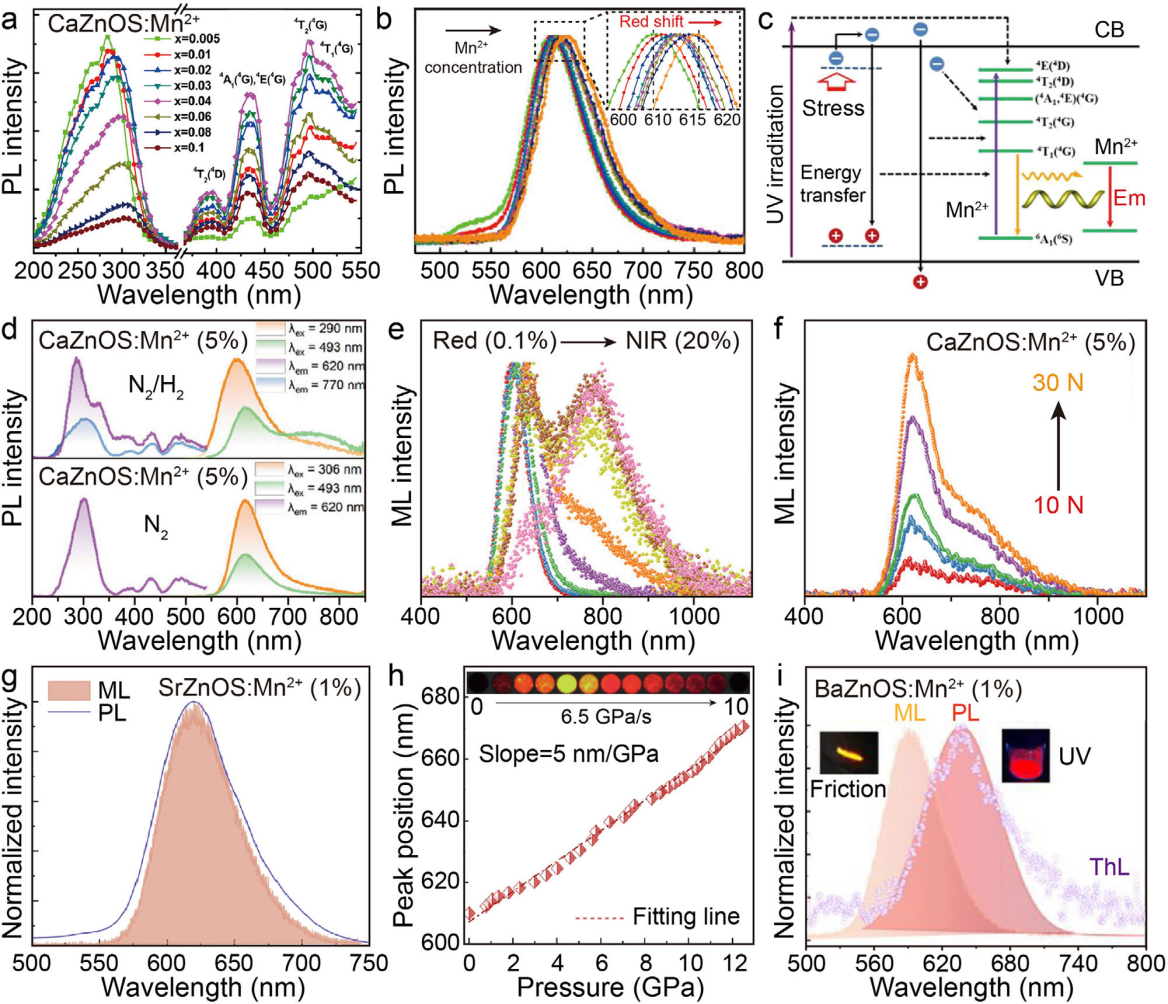
a) Excitation and b) the normalized emission spectra of CaZnOS: Mn^2+^ as a function of doping concentration from 0.5 to 10%. Inset of (b) shows the red‐shift. c) Schematic illustration of the multimode excited luminescence processes in CaZnOS: Mn^2+^. Reproduced with permission.^[^ ^34^ ^]^ Copyright 2015, American Chemical Society. d) Excitation and emission spectra of CaZnOS: Mn^2+^ sintered under N_2_/H_2_ (top) and N_2_ (bottom) conditions. e) The normalized ML spectra of CaZnOS: Mn^2+^ sintered under N_2_/H_2_ as a function of doping concentration from 0.1 to 20%. f) Relationship between the ML intensity of the sample and the applied force. Reproduced with permission.^[^ ^48^ ^]^ Copyright 2025, Wiley‐VCH. g) Comparison of the ML and PL spectra (*λ*ex = 375 nm) in SrZnOS: Mn^2+^ at ≈1.0 GPa. h) Peak position of ML as a function of pressure. The data were fitted linearly with a slope of 5.0 nm/GPa. Inset is the image of ML under compression at rates of 6.5 GPa/s at room temperature. Reproduced with permission.^[^ ^59^ ^]^ Copyright 2025, Nature. i) Comparison of the ML (orange), PL (red), and ThL (purple cycle) spectra of BaZnOS: Mn^2+^. Insets are ML and PL optical images induced by the friction and UV stimuli, respectively. Reproduced with permission.^[^ ^95^ ^]^ Copyright 2025, Wiley‐VCH. All spectra except the ThL spectrum were obtained at room temperature.

The PL properties of Mn^2+^ in the AZnOS mainly arise from the symmetry of its 3*d* ^5^ electronic configuration, permitting *d*–*d* transitions and spin‐forbidden effects, which result in its emission spectrum usually appearing stably in the orange–red light region (580–650 nm).^[^ ^43^, ^92^ ^]^ In an earlier study, Hintzen et al. observed a symmetric emission band centered at 614 nm (^4^ *T*_1_ → ^6^ *A*_1_) in CaZnOS: Mn^2+^ and explicitly attributed its red emission to the *d*–*d* transition process of Mn^2+^ under the action of the lattice field.^[^ ^40^ ^]^ Further, Huang et al. revealed the microscopic mechanism of the red‐light emission in this system by density‐functional theory (DFT) calculations, pointing out that the defect‐induced *Kohn*‐*Sham* single‐particle energy level difference in different host materials is the key factor determining the emission wavelength.^[^ ^92^ ^]^ Notably, the Mn^2+^ doping concentration has a significant modulation effect on the emission characteristics. When the doping concentration increases, the emission peak position of CaZnOS: Mn^2+^ is red‐shifted in the range of 600–630 nm (Figures 8a and b).^[^ ^34^ ^]^ PL spectral analysis indicates that as Mn^2+^ concentration increases, both the excitation peak of the host lattice and the characteristic emission peak of Mn^2+^ exhibit a tendency to shift toward longer wavelengths, accompanied by an enhancement of the Mn^2+^ self‐activation effect. Zhang et al. pointed out that when Mn^2+^ forms coordination pairs in the lattice, the energy gap between its ground state and the first excited state decreases, resulting in the ion‐pair emission energy being lower than that of the individual ions, which triggers a red‐shift of the emission wavelength (**Figure** **8**c).^[^ ^40^, ^43^ ^]^ The recent breakthrough was realized by Liu's research group: by precisely tuning the Mn^2+^ doping concentration (0.1–20.0%) and the synthesis atmosphere, the continuous tuning of the bimodal emission of PL and ML from the red to the NIR region has been achieved for the first time in the CaZnOS: Mn^2+^.^[^ ^48^ ^]^ In the 5.0% Mn^2+^ doped sample prepared under N_2_/H_2_ mixed atmosphere, the PL spectrum (top of Figure 8d) shows a new NIR emission band at 770 nm compared to the N_2_ atmosphere synthesized sample (bottom of Figure 8d), and this characteristic peak is also observed in the ML spectrum (Figure 8e). As the Mn^2+^ concentration exceeds 5%, the percentage of red emission intensity tends to decrease linearly, while the intensity of the NIR emission peak at 770 nm increases significantly. Liu et al. propose that this red‐shift phenomenon of the emission peaks originates from the enhanced magnetic dipole interactions between the Mn^2+^ ion pairs, which is further strengthened by the additional lattice defects introduced under the calcination in a reducing atmosphere.^[^ ^93^ ^]^ It is worth emphasizing that this NIR emission is highly sensitive to external pressure stimuli (Figure 8f), and pressure loading experiments revealed the simultaneous presence of dual luminescence centers originating from isolated Mn^2+^ (≈616 nm) and its pairs (≈770 nm) in the highly doped samples. The work by Gan et al. is equally noteworthy.^[^ ^94^ ^]^ They synthesized CaZnOS: Mn^2+^ using Mn^4+^‐based oxides as dopant precursors. During calcination, Mn^4+^ undergoes spontaneous reduction, forming defect complexes within CaZnOS. This process significantly induces host lattice distortion, enhancing the internal piezoelectric response. By employing this simple self‐reduction strategy based on defect‐induced mechanisms, Gan et al. effectively enhanced the ML properties of CaZnOS: Mn^2+^. These studies not only deepen our understanding of luminescence modulation through dopant concentration control and defect engineering but also provide a scalable approach for designing next‐generation phosphors and smart light‐emitting devices.

The SrZnOS: Mn^2+^ exhibits highly similar PL properties to the CaZnOS: Mn^2+^, which results from the fact that Mn^2+^ occupies the same coordination environment of the Zn^2+^ site (Figure 2). Based on this structural similarity, Zhao et al. pointed out that the layered lattice structure of SrZnOS can effectively promote the spatial separation and migration of electron–hole pairs, which results in excellent carrier transport properties during both PL and ML processes.^[^ ^56^ ^]^ In a recent study, Yang et al. carried out a systematic study on the pressure‐responsive ML properties of SrZnOS: Mn^2+^, focusing on the self‐recovered luminescence behavior under different strain rate conditions.^[^ ^59^ ^]^ As shown in Figure 8g, in the low‐pressure stage (≤1 GPa), the ML spectra of SrZnOS: Mn^2+^ are in high agreement with the PL spectra, which both show emission peaks centered at 615 nm. With the continuous increase of applied pressure, an emission peak red‐shift phenomenon similar to that of the CaZnOS: Mn^2+^ was experimentally observed (Figure 8h), and this wavelength shift can be attributed to the enhancement of the crystal field strength due to the high‐pressure environment, which in turn induces a narrowing of the *d*–*d* energy level spacing of Mn^2+^.^[^ ^96^ ^]^ It is particularly noteworthy that the luminescence intensity of SrZnOS: Mn^2+^ exhibits rate‐dependent oscillations in dynamic loading experiments: when compressed at a rate of 0.6 GPa/s, the sample exhibits a periodic luminescence of alternating light and dark, whereas when the loading rate is elevated to 6.5 GPa/s, the oscillation period is shortened and the intensity of luminescence is enhanced (inset of Figure 8h). Yang et al. confirmed by in situ high‐pressure synchrotron radiation experiments that this anomalous luminescence behavior originates from the enhanced carrier interaction between the host lattice and dopant ions under high‐pressure conditions, and that this interaction mechanism makes it potentially valuable for applications in the field of stress sensing.^[^ ^97^ ^]^ Since the first report of the PL properties of the BaZnOS: Mn^2+^ by Shi et al. in 2008, the progress of this system has been relatively lagging behind, which is mainly attributed to its significantly weaker luminescence performance than that of the Ca(Sr)ZnOS: Mn^2+^.^[^ ^51^ ^]^ It was not until 2016 that Peng's group systematically characterized the ML properties of this system for the first time, revealing that a weak emission band with the luminescence center located at ≈610 nm could only be observed under high‐pressure conditions (>1000 N). Until recently, Yang's team demonstrated in separate work that BaZnOS: Mn^2+^ also exhibits strong, reproducible, and self‐recovering piezoelectric activation of ML emission at GPa levels (Figure 8i).^[^ ^95^ ^]^ They demonstrated through thermoluminescence (ThL) curves the existence of defect traps with depths of 0.7–0.82 eV in BaZnOS. Upon Mn^2+^ doping, local crystal asymmetry emerged, ultimately revealing self‐recovering ML in BaZnOS: Mn^2+^ under strong external force. Despite its weak luminescence intensity and harsh excitation conditions, this unique pressure quenching effect provides a theoretical reference for the design of novel stress threshold sensors.

**Figure 9:**
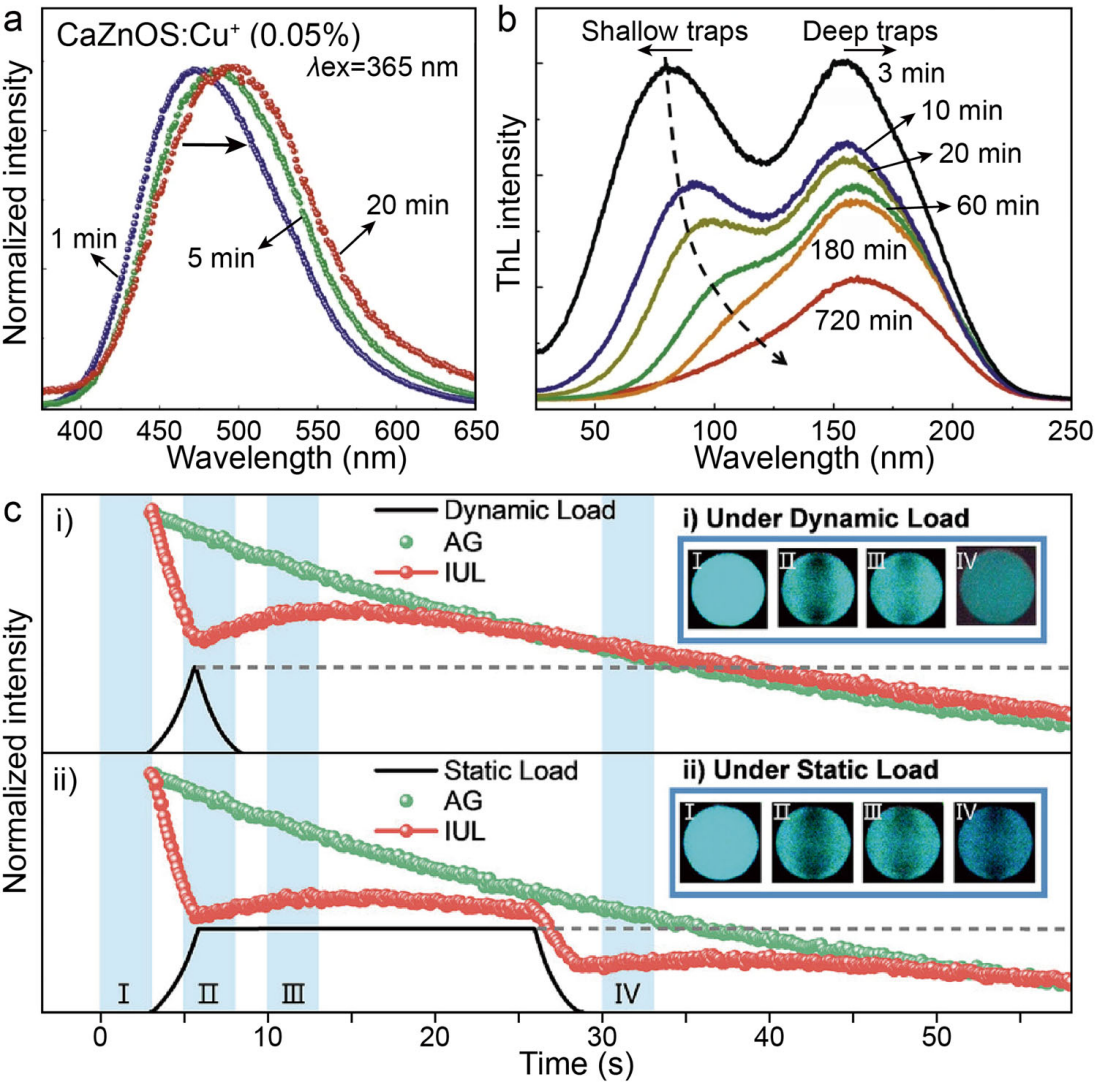
a) Phosphorescence spectra recorded 1, 5, and 20 min after stopping the irradiation in CaZnOS: Cu^+^. b) ThL curves at different decay times (3, 10, 20 min and 1, 3, 12 h) monitored at 472 nm from 25 to 250 °C of CaZnOS: Cu^+^. Reproduced with permission.^[^ ^44^ ^]^ Copyright 2015, Nature. c) ML intensity with force under dynamic and static load in CaZnOS: Cu^+^. The inserted photos correspond to the four stages in the diagram: i) under dynamic load, ii) under static load. Reproduced with permission.^[^ ^102^ ^]^ Copyright 2025, Wiley‐VCH.

Cu^+^ is usually used as a co‐dopant to mediate energy transfer in inorganic luminescent materials, for example, in the Cu^+^/Ln^3+^ co‐doping system, its mechanism is mainly manifested in the construction of additional donor–acceptor (*D*–*A*) energy level pairs, which can effectively modulate the energy resonance transfer process between the host and the Ln activator.^[^ ^98^ ^]^ However, Huang et al. revealed that deep energy‐level hole trap centers are formed when singly doped Cu^+^ replace the Zn^2+^ lattice sites, resulting in the host exhibiting significant long afterglow luminescence properties.^[^ ^92^, ^99^ ^]^ In particular, the mechanical quenching (MQ) phenomenon of phosphorescence intensity decay is observed when mechanical stress is applied to Cu^+^ doped samples, which provides a new perspective to understand the mechanism of ML. The work of Tu et al. in 2015 pointed out that CaZnOS: Cu^+^ samples were excited by UV light (365 nm) with continuous excitation, the phosphorescence emission peak of CaZnOS: Cu^+^ showed a red‐shift phenomenon with the prolongation of irradiation time (**Figure** **9**a).^[^ ^44^, ^100^ ^]^ This phenomenon was attributed to the enhancement process of the donor‐recipient complex effect in the host material. ThL analysis further confirmed the existence of 0.42 eV (shallow traps) and 0.71 eV (deep traps) double trap energy level structures in the CaZnOS: Cu^+^. With the increase of decay time, the shallow trap energy level carriers are gradually released, while the deep trap energy level carriers are retained (Figure 9b). This energy level distribution property leads to a unique ML behavior: during the initial stress loading, the carriers trapped in the shallow trap annihilate through the non‐radiative composite pathway, triggering the MQ effect; the continuous pressure application drives the release of electrons from the deep energy level, generating the ML signal.^[^ ^101^ ^]^ In recent research progress, Tu and coworkers integrated CaZnOS: Cu^+^ with the self‐developed mechanical switching device, and successfully constructed a stress–luminescence intensity encoding system by real‐time monitoring of the dynamic change of luminescence intensity during stretching.^[^ ^102^ ^]^ The system can realize the precise distinction between dynamic stress loading and static stress holding states, providing an innovative solution for static stress sensing under microstrain stimulation (Figure 9c). This material‐device co‐design strategy not only expands the application dimension of ML materials but also opens up an important technological path for the development of new smart sensing interfaces.

**Figure 10:**
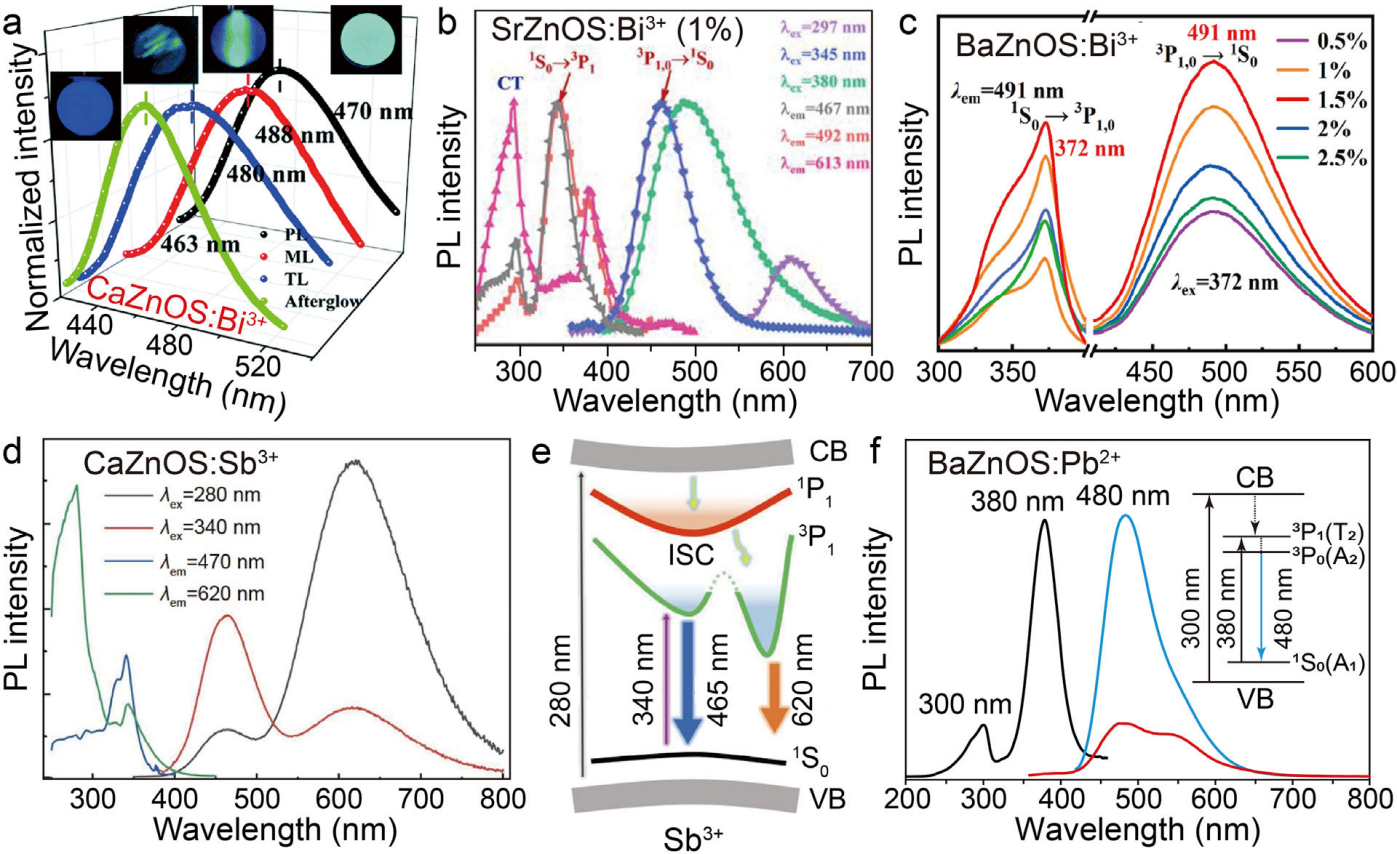
a) Spectra and images of PL (*λ*ex = 290 nm), ML (load = 5000 N), TL (load = 3 N), and afterglow of CaZnOS: Bi^3+^, respectively. Reproduced with permission.^[^ ^106^ ^]^ Copyright 2020, The Royal Society of Chemistry. b) The normalized excitation and emission spectra of SrZnOS: Bi^3+^ with *λ*ex = 297, 345, 380 nm, and monitoring *λ*em = 462, 492, 613 nm. Reproduced with permission.^[^ ^104^ ^]^ Copyright 2022, American Chemical Society. c) Excitation and emission spectra of the BaZnOS: Bi^3+^ under *λ*ex = 372 nm and *λ*em = 491 nm. Reproduced with permission.^[^ ^54^ ^]^ Copyright 2023, Elsevier. d) Excitation and emission spectra of the CaZnOS: Sb^3+^ under *λ*ex = 280, 340 nm and *λ*em = 470, 620 nm, and the corresponding e) schematic diagram of the charge transfer mechanism. Reproduced with permission.^[^ ^110^ ^]^ Copyright 2022, Science China. f) Excitation and emission spectra of BaZnOS: Pb^2+^ under *λ*ex = 300, 380 nm and *λ*em = 480 nm. Inset shows the energy level diagram of Pb^2+^. Reproduced with permission.^[^ ^58^ ^]^ Copyright 2022, Wiley‐VCH. All spectra were obtained at room temperature.

Main‐group elemental ions, such as Bi^3+^, Sb^3+^, and Pb^2+^, exhibit unique physicochemical properties in the field of luminescent materials due to their versatile valence characteristics and strong interactions with the host lattice. Among them, Bi^3+^ exhibits bifunctional properties in the AZnOS, acting both as a luminescent center (activator) and an energy transfer medium (sensitizer), and has received extensive attention in recent years.^[^ ^54^, ^103^, ^104^, ^105^ ^]^ In particular, it is noteworthy that the 6*s* ^2^6*p* electronic configuration of Bi^3+^ is highly sensitive to the crystal field environment, which allows it to exhibit broad spectral response properties from the UV to the red region in different hosts, and to achieve tunable luminescent color output. For example, in the CaZnOS: Bi^3+^, different excitation modes can yield green to blue emission wavelength variations (**Figure** **10**a).^[^ ^106^ ^]^ Compared to the PL spectra, the emission peaks of its ML and triboluminescence (TL) spectra show a significant red‐shift, while the afterglow spectra show the opposite blue‐shift phenomenon. Zhang et al. pointed out that this emission peak shift results from the strong dependence of the luminescence properties of Bi^3+^ on the crystal field environment. When the crystal field is disturbed by the external environment, the radiative intensity ratios of the ^3^ *P*_0,1_ → ^1^ *S*_0_ of Bi^3+^ are changed, which leads to the luminescence peak shift.^[^ ^107^, ^108^ ^]^ It was further shown that in the SrZnOS: Bi^3+^ (Figure 10b), the characteristic emission peak at 467 nm results from the synergistic excitation of the strong broad absorption band 345 nm (corresponding to the ^1^ *S*_0_ → ^3^ *P*_1_ of Bi^3+^) and the weak absorption band at 297 nm (host absorption).^[^ ^104^ ^]^ Notably, emission peaks induced by host absorption (located at 297, 345, 380 nm) were also observed at 613 nm. When the characteristic absorption peak of the BaZnOS host (located at 372 nm) is employed to excite the BaZnOS: Bi^3+^, the characteristic emission located at 491 nm is obtained (Figure 10c).^[^ ^54^ ^]^ With the increase of Bi^3+^ doping concentration, the emission quenching effect gradually appears. The above phenomenon indicates that the emission properties of Bi^3+^ in the AZnOS are highly sensitive to the lattice characteristics (e.g., bond length, coordination number, symmetry).^[^ ^103^, ^105^, ^109^ ^]^ This structure–property correlation provides important insights for the design of novel luminescent materials, i.e., the precise modulation of the emission properties of Bi^3+^ can be realized by tuning the microenvironment of the host lattice. Based on this tunable luminescence property, AZnOS: Bi^3+^ show remarkable potential for applications in areas such as *pc*‐LEDs lighting devices and stress visualization sensing, and in particular, their multimodal luminescence response properties provide new research paths for the development of a new generation of smart optical materials.

Compared with other main‐group elemental ions, Sb^3+^ exhibits unusually rich photophysical properties due to its unique 5*s* ^2^ electronic configuration. The coupling effect between this sterically active electronic structure and the lattice environment enables Sb^3+^ to commonly serve as a key luminescent center or co‐dopant in specific inorganic luminescent systems such as metal halides.^[^ ^111^, ^112^ ^]^ However, in AZnOS, the luminescence efficiency of Sb^3+^ is limited by the strong structural confinement effect of the host lattice, resulting in a relatively limited number of related research reports.^[^ ^113^ ^]^ Notably, the previous work of our group revealed the special luminescence properties of the CaZnOS: Sb^3+^ (Figure 10d): its emission spectrum exhibits dual emission bands characterized by 465 nm (blue) and 620 nm (orange), which can be cooperatively excited by both the host absorption peaks at 280 nm and the Sb^3+^ absorption peaks at 340 nm.^[^ ^110^ ^]^ Spectral analysis shows that these emissions result from the transition process between the ground state (^1^ *S*_0_) and the singly and triply excited states (^1^ *P*_1_) of Sb^3+^ (Figure 10e), and there is an obvious ^1^ *P*_1_ → ^3^ *P*_1_ inter‐system scampering channel. By regulating the excitation wavelength, the color shift from orange to blue light can be achieved, and combined with its ultrawide emission band (full width at half maxima of ≈136 nm), the CaZnOS: Sb^3+^ shows potential application value in the field of solid‐state lighting and multimode display.

As a typical *s* ^2^ electronic configuration ion, Pb^2+^ has unique energy level structure characteristics in inorganic luminescent materials. The first excited state of its *s*–*p* group state splits into three triplet states (^3^ *P*_0,1,2_) and one singlet state (^1^ *P*_1_) through spin–orbit coupling, and this energy level layout provides it with a broad‐spectrum emission capability covering the blue to red light region.^[^ ^114^, ^115^ ^]^ Taking BaZnOS: Pb^2+^ as an example (Figure 10f), the 300 nm host absorption peak together with the characteristic excitation peak of Pb^2+^ at 380 nm (^1^ *S*_0_ → ^3^ *P*_1_) induce the generation of broad‐spectrum emission at 480 nm, which results from ^3^ *P*_0_/^1^ *P*_1_ → ^1^ *S*_0_ transitions.^[^ ^58^ ^]^ Although Pb^2+^ exhibits excellent luminescent properties, its ecotoxicity as a typical heavy metal ion severely limits practical applications. Even trace exposures can cause irreversible damage to ecosystems and human health through bioaccumulation effects, which has prompted researchers to turn to the development of other nontoxic ions with similar *s* ^2^ electronic configurations.^[^ ^116^ ^]^

### 3.3 Mixed ion doping and sensitized luminescence

AZnOS doped with Ln, TM, and other main‐group element ions have shown significant potential for luminescence applications due to their tunable emission spectra across the VIS to NIR wavelength bands. However, the emission efficiency of a single‐doped system is often reduced due to various mechanisms, typically including inefficient excitation of Ln due to the parity check forbidden nature of the electron leap. To overcome these limitations, researchers often employ a multi‐ion co‐doping strategy to construct hierarchical energy level structures introduced by different dopant ions in the AZnOS semiconductor bandgap.^[^ ^87^, ^98^ ^]^ These energy levels can be optimally designed for the luminescence process by modulating the excitation conditions. Based on this, this review systematically combed several types of typical co‐doping systems, and analyzed their luminescence properties, energy transfer mechanisms, and luminescence behaviors under multimode excitation, with a view to providing theoretical references for the design of AZnOS based luminescent materials.

**Figure 11:**
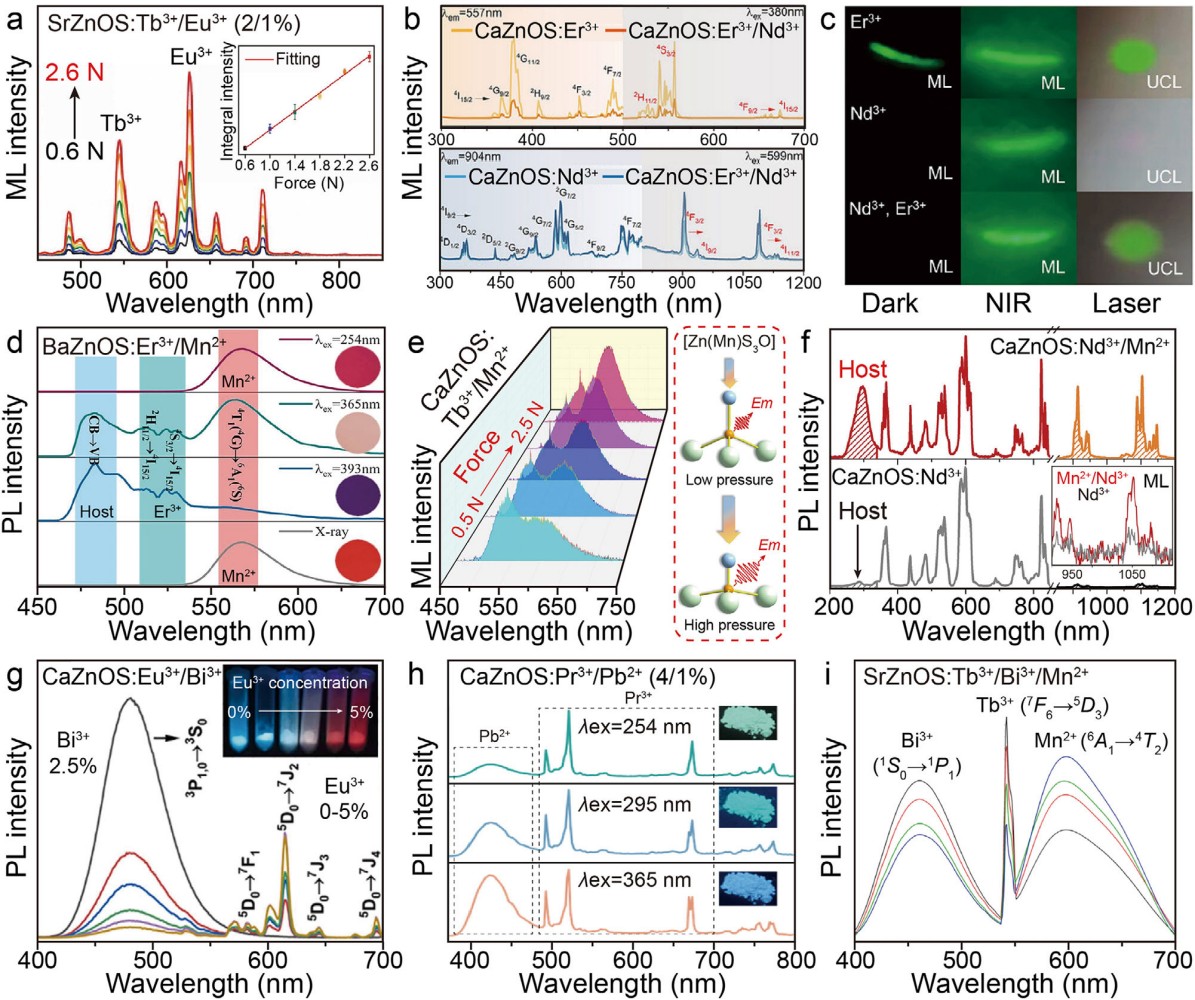
a) ML spectra of the SrZnOS: Tb^3+^/Eu^3+^ under different loading forces from 0.6 to 2.2 N. Inset shows integral intensity of ML under different loading forces. Reproduced with permission.^[^ ^57^ ^]^ Copyright 2021, Wiley‐VCH. b) Excitation and emission spectra of CaZnOS: Er^3+^and CaZnOS: Er^3+^/Nd^3+^ (top, *λ*ex = 380 nm, *λ*em = 557 nm), CaZnOS: Nd^3+^ and CaZnOS: Er^3+^/Nd^3+^ (bottom, *λ*ex= 599 nm, *λ*em= 904 nm), respectively. c) ML and UCL images of CaZnOS: Er^3+^, CaZnOS: Nd^3+^ and CaZnOS: Er^3+^/Nd^3+^, respectively. Reproduced with permission.^[^ ^119^ ^]^ Copyright 2025, Wiley‐VCH. d) Emission (*λ*ex= 254, 365, 393 nm) and radioluminescence (X‐ray= 5 mGy S^−1^) spectra of BaZnOS: Er^3+^/Mn^2+^. Reproduced with permission.^[^ ^120^ ^]^ Copyright 2022, Elsevier. e) Normalized ML spectra of CaZnOS: Tb^3+^/Mn^2+^ under different loading forces (left), and the variation of tetrahedron structure of [Zn(Mn)S_3_O] and the corresponding variation in ML intensity with different pressures (right). Reproduced with permission.^[^ ^121^ ^]^ Copyright 2022, Wiley‐VCH. f) Excitation and emission spectra of CaZnOS: Nd^3+^/Mn^2+^ (top) and CaZnOS: Nd^3+^ (bottom) (*λ*ex= 277 nm, *λ*em= 914 nm), respectively. Inset is the compression‐induced ML spectra of these two samples. Reproduced with permission.^[^ ^122^ ^]^ Copyright 2023, The Royal Society of Chemistry. g) Emission spectra of CaZnOS: Eu^3+^/Bi^3+^ as a function of Eu^3+^ doping concentration. Inset is the photo of the samples under 365 nm excitation. Reproduced with permission.^[^ ^109^ ^]^ Copyright 2022, Elsevier. h) Emission spectra and the corresponding photographs of the CaZnOS: Pr^3+^/Pb^2+^ under *λ*ex= 254, 295, and 365 nm. Reproduced with permission.^[^ ^123^ ^]^ Copyright 2024, American Chemical Society. i) Emission spectra of SrZnOS: Tb^3+^/Bi^3+^/Mn^2+^ under *λ*ex= 350 nm. Reproduced with permission.^[^ ^124^ ^]^ Copyright 2022, Elsevier. All spectra were obtained at room temperature.

To improve the luminescence sensitization efficiency of Ln, a typical strategy is to achieve energy level modulation by co‐doping a variety of Ln with permissive electron leap properties. Taking the Ce^3+^/Tb^3+^ co‐doped system as an example, Ce^3+^ can effectively enhance the absorption of UV light by the host, and subsequently, through the energy transfer from the excited state Ce^3+^ to Tb^3+^, it significantly improves the Tb^3+^ luminescence efficiency.^[^ ^117^, ^118^ ^]^ In recent years, the co‐doping system of Tb^3+^ (^5^ *D*_4_ → ^7^ *F*_5_, green emission) and Eu^3+^ (^5^ *D*_0_ → ^7^ *F*_2_, red emission) co‐doped systems has shown unique advantages in the field of stress–temperature dual‐parameter sensing due to the coexistence of double independent emission peaks. For typical cases, the SrZnOS: Tb^3+^/Eu^3+^ phosphor reported by Xie's team (**Figure** **11**a) realized a linear positive correlation between the ML integral intensity and the stress magnitude, and the *I*_Tb_/*I*_Eu_ intensity ratio shows a temperature‐dependent linear response with increasing stress.^[^ ^57^ ^]^ Based on this property, the researchers constructed a stress–temperature bimodal imaging system, and achieved two‐parameter visual sensing with a maximum relative sensitivity (*S_r_*_max_) of 0.877% K^−1^ in the 298–473 K temperature range. However, the luminescence bands of the current dual Ln^3+^‐doped system are mostly concentrated in the visible region, and there are two key problems: first, the adjacent or overlapping luminescence bands lead to the difficulty of decoupling the multi‐physical parameters; and second, the visible band is susceptible to the interference of ambient light.^[^ ^48^ ^]^ Therefore, the development of AZnOS‐based luminescent materials in the NIR spectral region is of great research value. In the latest research progress, Chen et al. successfully synthesized CaZnOS: Nd^3+^/Er^3+^, realizing the synergistic effect of the highly efficient NIR ML of Nd^3+^ with the UCL of Er^3+^ (Figure 11b).^[^ ^119^ ^]^ Spectral analysis showed that the introduction of Nd^3+^ suppresses the characteristic emission of Er^3+^, while the presence of Er^3+^ enhances the luminescence intensity of Nd^3+^, and the lifetime decay curves confirm the existence of an energy transfer channel between them. Finally, the NIR ML phenomenon was observed simultaneously with UCL emission under 980 nm excitation in the same sample (Figure 11c). The above study provides an important experimental basis for expanding the applications of AZnOS based materials in multimodal luminescence and non‐contact sensing.

Current studies consistently show that the Ln^3+^‐doped AZnOS have a significant host sensitization effect. However, Zhang et al. found significant differences in the sensitization efficiency of different Ln in CaZnOS, Tm^3+^, Dy^3+^, Ho^3+^, Er^3+^, and Nd^3+^ excitation spectra were mainly dominated by the characteristic *f*–*f* transition peaks of Ln, indicating that the energy transfer efficiency from host to Ln was low.^[^ ^98^ ^]^ To enhance the energy transfer efficiency, researchers have employed a co‐doping strategy to introduce luminescent ions with different electron leap properties. Such co‐doping not only regulates the energy resonance between the host and Ln by forming additional *D*–*A* energy levels but also serves as an energy mediator to facilitate the transfer process. For example, Zhang et al. observed in the CaZnOS: Ln^3+^/Cu^+^ that co‐doped Cu^+^ enhances the excitation band of the host and achieves efficient luminescence of Ln by efficiently trapping the lattice excitation energy.^[^ ^98^ ^]^ Inspired by bandgap engineering, the Ln^3+^/Mn^2+^ co‐doping strategy was used to optimize the AZnOS. It was found that Mn^2+^ could reduce the bandgap and effectively improve the energy transfer path from host to Ln. This strategy has been successfully realized in visible‐emitting ion‐pair co‐doping systems such as Er^3+^/Mn^2+^,^[^ ^125^ ^]^ Tb^3+^/Mn^2+^,^[^ ^58^ ^]^ and Ho^3+^/Mn^2+^.^[^ ^121^ ^]^ In the case of BaZnOS: Er^3+^/Mn^2+^ reported by Yu et al. (Figure 11d), the characteristic Mn^2+^ emission is observed at 254 nm UV and X‐ray excitation; at 393 nm excitation, the bandgap (470 nm) with Er^3+^'s ^2^ *H*_11/2_/^4^ *S*_3/2_ → ^4^ *I*_15/2_ (525/550 nm) transitions at the same time; and 365 nm excitation then triple emission of bandgap, Er^3+^, and Mn^2+^ is realized.^[^ ^120^ ^]^ A similar phenomenon is also verified in the CaZnOS: Er^3+^/Mn^2+^.^[^ ^87^ ^]^ This multimode excitation tunable luminescence strategy has the advantages of simplicity and reproducibility, and provides a new idea for optical tuning and coding. Wang and co‐workers put forward another viewpoint: the *d*–*d* transition of TM is highly sensitive to the local crystal field changes due to the susceptibility of the outermost 3*d* electrons to environmental influences; whereas, the 4*f* orbitals of Ln are shielded by the 5*s*25*p* ^6^ outer electrons, and its *f*–*f* transition is relatively insensitive to the crystal field changes.^[^ ^121^ ^]^ Taking the CaZnOS: Tb^3+^/Mn^2+^ as an example, the excitons in the defect energy levels under stress are proportionally assigned to Tb^3+^ and Mn^2+^. Stress‐induced lattice contraction or distortion enhances the local crystal field, resulting in a radiative relaxation rate of the Mn^2+^ coordination environment that exceeds that of Tb^3+^, which ultimately results in a higher luminescence intensity for Mn^2+^ than for Tb^3+^ (Figure 11e).^[^ ^121^ ^]^ This strategy opens a new path for the design of stress‐responsive force‐emitting color‐tunable materials. In the NIR region, co‐doping of Nd^3+^ with Mn^2+^ can construct an energy transfer pathway of host→ Mn^2+^→ Nd^3+^, which significantly enhances the NIR ML performance of Nd^3+^ (^4^ *F*_3/2_ → ^4^ *I_11,_*_9/2_) (Figure 11f).^[^ ^122^ ^]^ This design enhances the phosphor's tissue penetration ability and obtains higher signal‐to‐noise ratio NIR luminescence images (inset of Figure 11f), advancing the development of CaZnOS: Nd^3+^/Mn^2+^ for in vivo insitu biomechanical imaging.

The ^3^ *P*_0_ → ^1^ *S*_0_ transition of Bi^3+^ exhibits a blue broad peak emission at ≈480 nm, making it an ideal candidate for co‐doping with red‐emitting Ln^3+^ (e.g., Eu^3+^, Sm^3+^) with the potential to achieve white light emission. Taking the CaZnOS: Eu^3+^/Bi^3+^ as an example (Figure 11g), Wu et al. confirmed the cascade energy transfer mechanism of host→ Bi^3+^→ Eu^3+^: the Eu^3+^ excitation bands (^7^ *F*_0_ → ^5^ *D*_2_) overlap spectrally with the emission bands of Bi^3+^, leading to electron transfer and radiative reabsorption processes from Bi^3+^ to Eu^3+^.^[^ ^109^ ^]^ Experiments show that the intensity of its own luminescence continues to decrease with the increase of the co‐doping concentration of Bi^3+^, while the luminescence intensity of Eu^3+^ is significantly enhanced. Through the concentration‐dependent quenching effect of Bi^3+^, the emission color of CaZnOS: Eu^3+^/Bi^3+^ can be linearly tuned from cyan via white to orange–red (inset of Figure 11g). Similarly, co‐doping of the Pb^2+^ with Pr^3+^ exhibits distinctive optical properties. Huang's team observed in the CaZnOS: Pr^3+^/Pb^2+^ that Pb^2+^→ Pr^3+^ energy transfer phenomenon, the mechanism of which results from the partial overlap of the excitation band of Pr^3+^ with the emission band of Pb^2+^.^[^ ^123^ ^]^ By adjusting the excitation wavelength, the emission color of this system can be gradually transformed from green (*λ*_ex_ = 254 nm) to cyan (*λ*_ex_ = 295 nm) and blue (*λ*_ex_ = 365 nm) (inset of Figure 11h). It was also noted that Pr^3+^/Pb^2+^ co‐doping introduces ultra‐shallow traps in the host, which trap UV‐excited carriers and release them slowly with changes in ambient temperature and storage time, affecting the luminescence kinetics.

Compared with the double‐doped systems, the studies on the triple‐doped systems are more limited, mainly due to the complex energy transfer pathways and unforeseen coupling effects that may be triggered by multi‐ion co‐doping.^[^ ^126^ ^]^ However, Sun et al. have recently realized bidirectional energy transfer from Bi^3+^→ Mn^2+^ and Tb^3+^→ Mn^2+^ in the SrZnOS: Tb^3+^/Bi^3+^/Mn^2+^. The system is characterized by blue (Bi^3+^: ^1^ *S*_0_ → ^3^ *P*_1_), green (Tb^3+^: ^7^ *F*_6_ → ^5^ *D*_3_), and red (Mn^2+^: ^6^ *A*_1_ → ^4^ *T*_2_) triple‐color emission overlays to obtain a bright white light output (Figure 11i). This multimodal luminescence modulation strategy provides a new idea for the design of *pc*‐LEDs.

### 3.4 Solid‐Solution Engineering and Multiphasic Assembly in the Host

**Figure 12:**
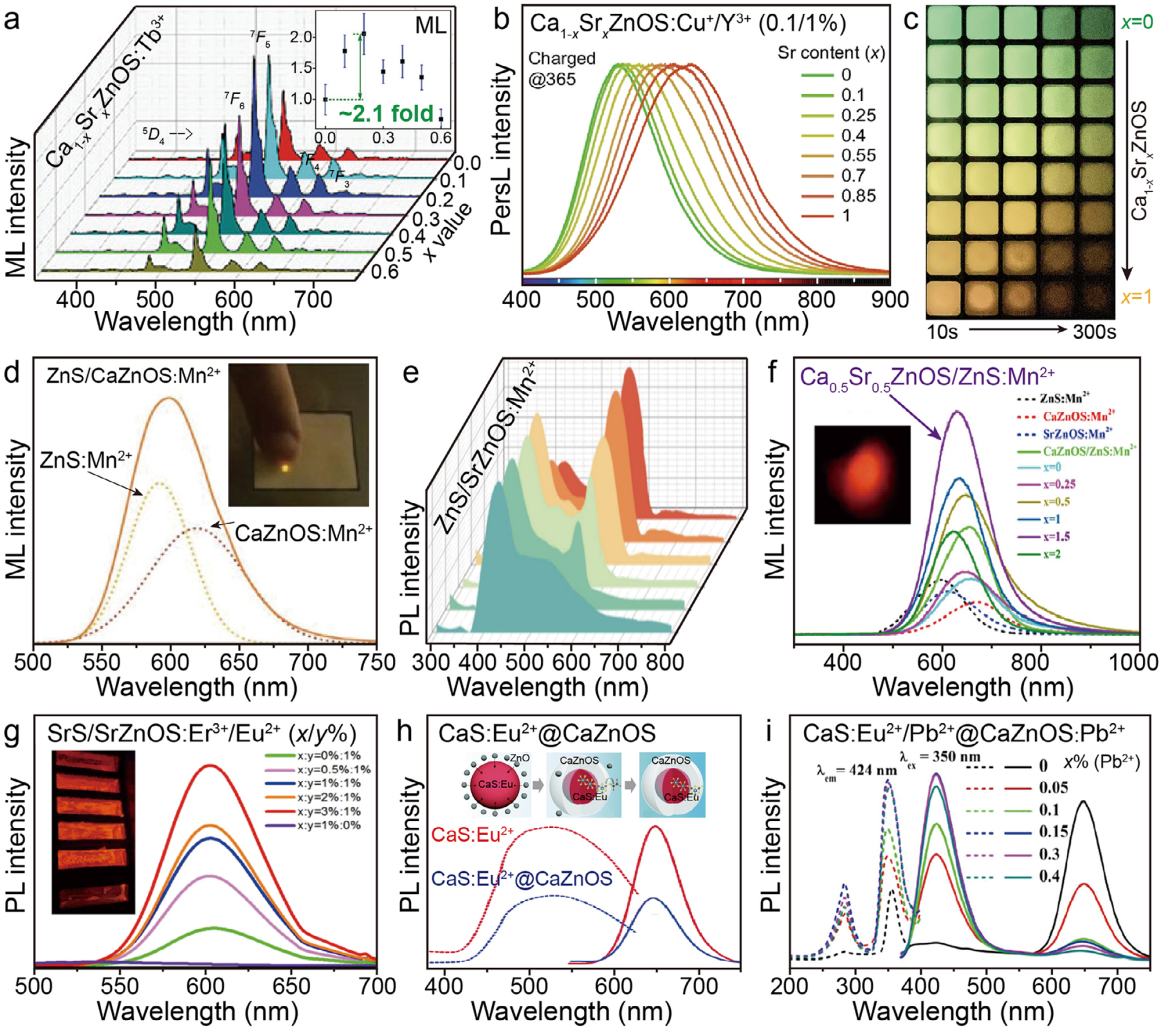
a) ML spectra of Ca_1‐_ *_x_* Sr*_x_* ZnOS: Tb^3+^ with increasing *x* value. Inset is the variation of the integrated ML intensities of the sample with increasing *x* value. Reproduced with permission.^[^ ^129^ ^]^ Copyright 2023, Wiley‐VCH. b) PersL spectra of Ca_1‐_ *_x_* Sr*_x_* ZnOS: Cu^+^/Y^3+^ (*x* = 0–1) after turning off the UV light (365 nm). c) PersL photographs of Ca_1‐_ *_x_* Sr*_x_* ZnOS: Cu^+^/Y^3+^ with various Sr^2+^ contents. Reproduced with permission.^[^ ^131^ ^]^ Copyright 2024, Nature. d) ML spectrum of the ZnS/CaZnOS: Mn^2+^. Inset is the photograph of the sample with the optimal composition under mechanical excitation with a finger. Reproduced with permission.^[^ ^45^ ^]^ Copyright 2020, Wiley‐VCH. e) Emission spectra of the ZnS/CaZnOS: Mn^2+^ at different Mn^2+^ doping concentrations. Reproduced with permission.^[^ ^132^ ^]^ Copyright 2024, Elsevier. f) ML spectra of ZnS: Mn^2+^, CaZnOS: Mn^2+^, SrZnOS: Mn^2+^, Ca_0.5_Sr_0.5_ZnOS/*x*ZnS: Mn^2+^ (*x* = 0–2), and CaZnOS/ZnS: Mn^2+^ obtained upon applying a 12 N contact load. Reproduced with permission.^[^ ^133^ ^]^ Copyright 2022, American Chemical Society. g) Emission spectra of SrS/SrZnOS: Er^3+^/Eu^2+^ under *λ*ex = 450 nm. Reproduced with permission.^[^ ^134^ ^]^ Copyright 2025, The Korean Institute of Metals and Materials. h) Excitation and emission spectra of CaS: Eu^2+^@CaZnOS and CaS: Eu^2+^ dispersed in 50 wt% acetic acid solution under *λ*ex = 550 nm. Inset is the schematic diagram of the formation of core–shell structured CaS@CaZnOS via inward erosion growth of a CaZnOS layer on the surface of CaS using ZnO, and the acidic washing treatment process to remove the residual ZnO. Reproduced with permission.^[^ ^135^ ^]^ Copyright 2019, The Royal Society of Chemistry. i) Excitation and emission spectra of CaS: Eu^2+^/Pb^2+^@CaZnOS: *x*Pb^2+^ (*x*= 0–0.4) under *λ*ex = 350 nm. Reproduced with permission.^[^ ^136^ ^]^ Copyright 2021, American Chemical Society. All spectra were obtained at room temperature.

Solid‐solution engineering can be regarded as a special doping strategy, the core of which lies in the precise regulation of the host lattice by means of ion substitution or compound mixing.^[^ ^127^ ^]^ Specifically, replacing specific ions in the native lattice with substitutes of similar chemical properties or comparable ionic radius to those in the host lattice, or fine‐tuning of the lattice parameters through the mixing of two isostructural compounds. The significant difference from conventional doping strategies is that solid‐solution engineering usually involves large‐scale ion substitution (the substitution ratio is usually more than 5%), whereas doping in luminescent materials tends to adopt low‐concentration doping strategies (the concentration is usually lower than 5% to avoid the concentration quenching effect). In the AZnOS, due to the highly consistent structural properties of CaZnOS and SrZnOS, researchers often utilize the Ca_1‐_ *_x_* Sr*_x_* ZnOS (*x* = 0–1) solid‐solution with linearly tunable physical properties by constructing the continuous variation of the Ca^2+^/Sr^2+^ ratio to achieve a highly controllable and linear shift to the *D*–*A* energy level. For example, Zhang et al. found that the Ca_0.7_Sr_0.3_ZnOS doped with Mn^2+^ enhances the ML intensity by about an order of magnitude compared to CaZnOS: Mn^2+^.^[^ ^128^ ^]^ However, the application of solid‐solution engineering may introduce lattice distortions or chemical stresses, which in turn promote the formation of intrinsic/exogenous defects, change the crystal field polarity, and ultimately lead to a change in the trap concentration or trap depth of the material. For example, Wang et al. showed that in Ca_1‐_ *_x_* Sr*_x_* ZnOS (*x* = 0–0.6), the concentration of oxygen vacancies in the host tends to increase as the proportion of Sr^2+^ substitution increases.^[^ ^129^ ^]^ The enhancement in trap depth implies an increase in the ability of the host to capture electrons, which is ultimately observed in Ca_0.8_Sr_0.2_ZnOS: Tb^3+^, with ≈2.1 times of ML intensity at ≈550 nm (^5^ *D*_4_ → ^7^ *F*_5_) compared to CaZnOS: Tb^3+^ (**Figure** **12**a). This study reveals a mechanism to modulate the oxygen vacancy concentration by cation substitution to enhance the luminescence performance of intrinsically defective phosphors. Notably, the persistent luminescence (PersL) phenomenon exhibits high sensitivity in such solid‐solutions with linearly tunable physical properties, and the precise tuning of PersL emission wavelength can be realized by designing the sub‐bandgap *D*–*A* energy level.^[^ ^130^ ^]^ For example, the Ca_1‐_ *_x_* Sr*_x_* ZnOS: Cu^+^/Y^3+^ (0.1/1%, *x* = 0–1) reported by Wang et al. exhibits significant PersL emission under 365 nm UV light charging (Figure 12b and c), and its PersL emission wavelength showed a linear red‐shift in the range of 500–630 nm, with a wavelength tuning accuracy of up to 5 nm.^[^ ^131^ ^]^ Theoretical calculations showed that the precise tuning of PersL wavelength resulted from the Ca^2+^/Sr^2+^ alloying effect on the band structure, i.e., the *D* energy level decreases with increasing alloying degree. This finding provides an innovative strategy for the fine‐tuning of luminescence properties and opens up new ideas for the design of multicolor luminescent materials.

Based on the inherent properties of the AZnOS as a wide‐bandgap piezoelectric semiconductor, effective modulation of the luminescence performance of the material can be realized by constructing a heterojunction structure consisting of two hosts.^[^ ^137^, ^138^, ^139^, ^140^ ^]^ This strategy opens up a new research path to enhance the ML performance of the host material. In view of the similarity between CaZnOS and ZnS crystal structures, Wang et al. innovatively prepared ZnS/CaZnOS heterojunction structures.^[^ ^45^ ^]^ It was found that the ML strength of this heterojunction was enhanced by 2.2 times compared to ZnS: Mn^2+^ and 3.5 times compared to CaZnOS: Mn^2+^ after doping with Mn^2+^ (Figure 12d). The enhancement mechanism can be attributed to the energy band shift effect at the heterojunction interface, which effectively reduces the excitation barriers of conduction‐band electrons, promotes the transport efficiency of interfacial carriers, and significantly improves the electron–hole complexation efficiency in the region adjacent to the luminescent ions.^[^ ^141^, ^142^, ^143^, ^144^ ^]^ In a subsequent study, Suchocki et al. successfully reconstructed ZnS/CaZnOS heterojunction using microwave‐assisted method and applied it to the field of electronic signature devices and security technology,^[^ ^145^ ^]^ while Bai et al. further developed ZnS/SrZnOS: Mn^2+^ heterojunction composites with high ML sensitivity (Figure 12e),^[^ ^132^ ^]^ which exhibits excellent stress distribution visualization properties with both high resolution and fast response time, providing important technical support for practical applications under low energy consumption conditions. Inspired by the study of dual host heterojunction, Zou et al. proposed a heterostructure building strategy based on a ternary host system.^[^ ^133^ ^]^ They successfully prepared by one‐pot method ternary Ca_0.5_Sr_0.5_ZnOS/1.5ZnS: Mn^2+^ heterojunction, which not only achieves a significant enhancement of the ML emission intensity but also exhibits a gradual blue‐shift of the wavelength in the 658–623 nm range (Figure 12f). This composite design strategy of ternary host and heterostructure provides an innovative paradigm for the development of new ML phosphor materials with higher efficiency. In another work, Zhang et al. observed dual‐mode luminescence properties in SrS/SrZnOS: Er^3+^/Eu^2+^ composites: the SrS exhibits orange–red PersL, while the SrZnOS component exhibits significant green ML (Figure 12g).^[^ ^134^ ^]^ Through the crystal structure synergistic effect, energy band configuration optimization, and precise tuning of the Er^3+^/Eu^2+^ luminescence centers, the composite achieves a significant enhancement in both long afterglow duration and ML intensity.

In view of the excellent thermal and chemical stability of the AZnOS as oxides, the researchers proposed the innovative strategy of using it as a surface coating agent, which can effectively solve the problem of performance degradation of traditional luminescent materials when exposed to moisture or heat.^[^ ^146^ ^]^ Based on this idea, Lian et al. successfully developed CaS: Eu^2+^@CaZnOS composite with a dense core–shell structure.^[^ ^135^ ^]^ The core–shell structure exhibited excellent stability to acid, alkali, water, and thermal environments, while the quantum efficiency in the green luminescence region was enhanced by up to 23% (Figure 12h). In the subsequent study, Lian et al. further introduced Pb^2+^ doping into the core and shell layers, and successfully prepared CaS: Eu^2+^/Pb^2+^@CaZnOS: Pb^2+^ composites, which achieved excellent dual excitation and emission luminescence performance (Figure 12i).^[^ ^136^ ^]^ In addition, TM such as Mn^2+^ and Cu^+^ were successively introduced into the core–shell structure, and the experiments showed that these dopants not only enhanced the blue light emission intensity, but also expanded the absorption range of UV light of the host, which demonstrated the great potential for the application in the field of agricultural light.

In conclusion, we systematically investigated the luminescent behavior of different dopant ions in AZnOS‐based phosphors, along with the optical properties of these phosphors under various influencing factors. Particularly noteworthy is the significant impact of environmental factors on the luminescent characteristics of AZnOS‐based phosphors, with ambient temperature being especially prominent.^[^ ^1^ ^]^ Elevated temperatures induce irreversible changes in the chemical composition, crystal structure, and defect density of phosphors, ultimately compromising their performance metrics in *pc‐LEDs* application, such as luminous efficiency, color stability, and luminous lifetime.^[^ ^147^ ^]^ Nevertheless, owing to its oxide nature, AZnOS exhibits exceptional high‐temperature stability.^[^ ^71^ ^]^ For instance, Zhang et al. confirmed via thermogravimetric analysis that CaZnOS maintains structural integrity without decomposition reactions across the temperature range from room temperature to ≈1100 K.^[^ ^82^ ^]^ However, excessively high temperatures significantly increase the non‐radiative transition probability of AZnOS‐based phosphors, thereby reducing their luminescence efficiency. For example, Liu et al. verified the temperature‐dependent luminescence characteristics of CaZnOS: Mn^2+^ through lifetime decay curves.^[^ ^48^ ^]^ Results indicate that lifetime values at room temperature are substantially lower than those at 80 K, stemming from the positive correlation between temperature and non‐radiative transition probability. Notably, Wang et al. observed enhanced luminescence in BaZnOS: Mn^2+^ from 303 to 403 K, attributed to enhanced thermal activation of energy transfer from defect states to Mn^2+^.^[^ ^58^ ^]^ However, temperatures beyond this range induce severe thermal quenching effects in BaZnOS: Mn^2+^. We believe that by combining the regulation of local coordination environments with strategic defect engineering design, it is possible to achieve effective and precise control over the energy levels of dopants and photophysical processes in AZnOS based phosphors. This integrated control strategy aims to establish a strong correlation between structure and properties, providing robust guidance for the development of advanced multifunctional luminescent materials.

## 4 Synthetic Protocols

**Figure 13:**
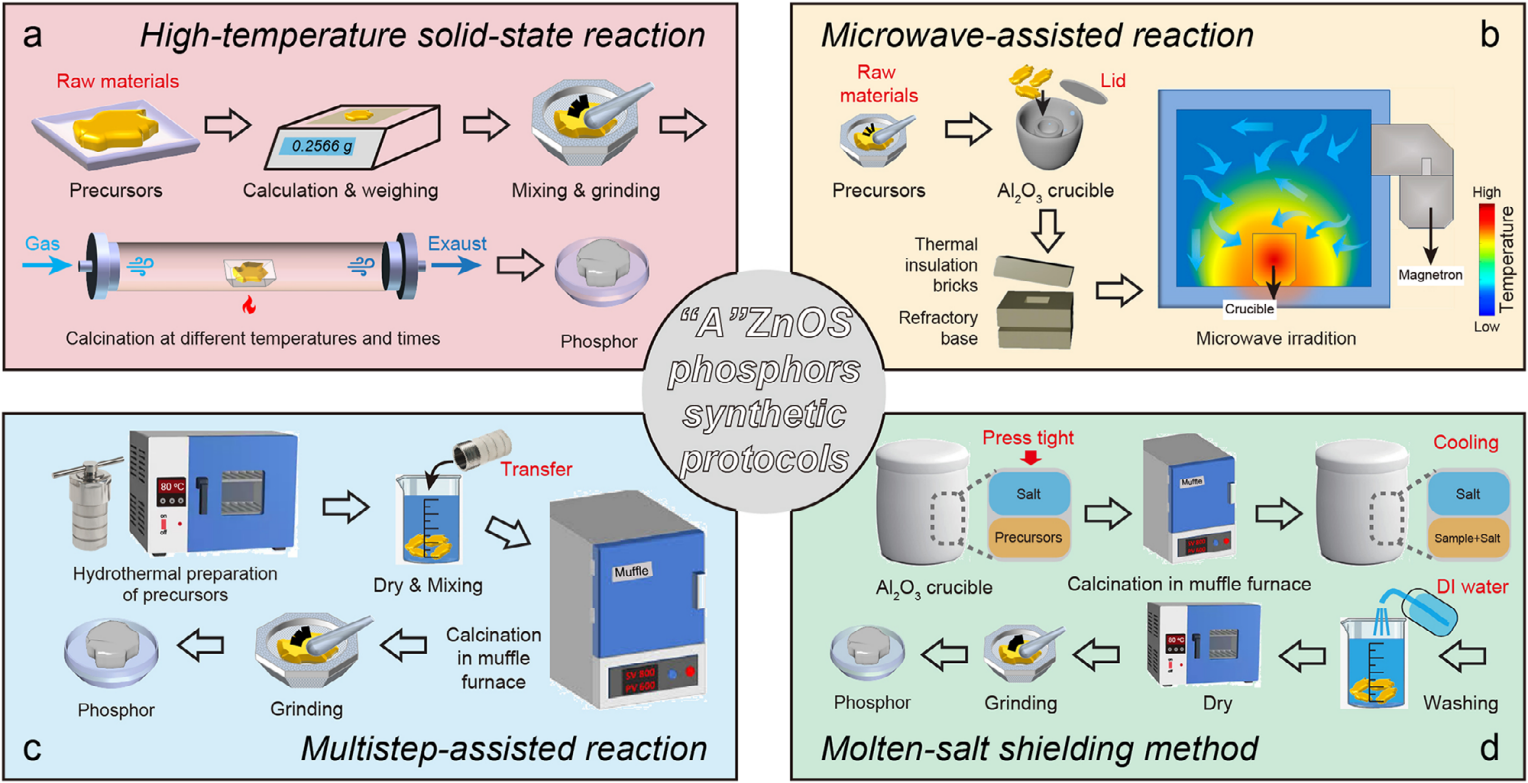
Schematic representation showing the preparation of AZnOS‐based phosphors using a) high‐temperature solid‐state reaction; b) microwave‐assisted reaction, which is reproduced with permission.^[^ ^148^ ^]^ Copyright 2021, MDPI; c) Multistep‐assisted reaction, and d) Molten‐salt shielding method, which is reproduced with permission.^[^ ^149^ ^]^ Copyright 2022, Wiley‐VCH.

In recent years, the AZnOS has become a research hotspot in the field of inorganic luminescent materials by virtue of its excellent PL and ML properties, and the related research has formed a more systematic research framework. By combing through the published articles, the synthesis strategies of this system can be summarized into the following four categories: high‐temperature solid‐state reaction, microwave‐assisted reaction, multistep‐assisted reaction, and molten salt shielding synthesis (**Figure** **13**). AZnOS prepared using the above process has been widely accepted by researchers due to its excellent crystallization properties and doping efficiency. However, these methods also face challenges such as complex synthesis processes and demanding conditions. Consequently, continuously optimizing reaction conditions to identify optimal and universally applicable solutions remains a critical challenge in the preparation of AZnOS.

Based on this, this section will systematically sort out the synthesis strategies of AZnOS, focus on analyzing the unique advantages of various methods in terms of crystallinity regulation, doping efficiency optimization, and morphology control, aiming to provide empirical references for the subsequent research and to promote the synthesis methods of this system towards cleanliness, economy, and safety.

### 4.1 High‐Temperature Solid‐State Reaction

In recent years, the research on the synthesis of AZnOS‐based phosphors mainly focuses on the traditional high‐temperature solid‐state reaction, which has the advantages of simple operation and good process reproducibility, and has become the mainstream technical route for the preparation of phosphors in this system.^[^ ^150^, ^151^ ^]^ The raw materials required for its synthesis mainly include carbonates (ACO_3_), oxides (AO), and zinc sulfide (ZnS) or other compounds, and luminescent ion doping is usually used as a precursor of RE oxides or decomposable compounds. The specific protocol is as follows: the mixed precursors are placed in a tube furnace and heated to 1000–1500 °C (Figure 13a) under the protection of inert atmosphere (such as N_2_ or Ar) for calcination; after the calcination is completed, the samples are cooled down to room temperature, and then milled, crushed, and sieved to obtain the AZnOS samples with homogeneous particle sizes.

For the synthesis of different AZnOS samples, the raw materials and reaction conditions are somewhat different. Taking CaZnOS as an example, its synthesis is usually based on CaCO_3_ and ZnS powders, which are dispersed in ethanol and then wet ground; the dried mixture is placed in an alumina crucible and sintered at a temperature of more than 1000 °C for over 3 h. The synthesis of BaZnOS is similar to that of CaZnOS, and the raw material CaCO_3_ can be replaced by BaCO_3_ only. In contrast, the synthesis of SrZnOS is more challenging: on one hand, its synthesis needs to be carried out within a narrow temperature window of ≈1050 °C; on the other hand, SrZn_2_OS_2_ multiphase impurities are highly prone to be generated during the synthesis process.^[^ ^53^, ^61^, ^62^ ^]^ Therefore, obtaining pure‐phase SrZnOS requires precise control of the reaction parameters to suppress the generation of impurity phases.

In the choice of raw materials for doping luminescent ions, it is more appropriate to use fluorides or oxides with their valence matching for the doping of Ln. This is because the two types of compounds not only have a lower melting point, but also can avoid the change of Ln valence state in the doping process, which is conducive to promoting the chemical reaction between raw materials. In addition, adding an appropriate amount of flux at the preparation stage is an effective means to optimize the material properties.^[^ ^58^, ^152^ ^]^ For example, adding a small amount of lithium salt (e.g., LiNO_3_, usually with a molar ratio of less than 2%) to the raw material for calcination can enhance the doping efficiency and improve the crystal crystallization quality, and the introduction of lithium salt can accelerate the ion diffusion and promote the entry of dopant ions into the host lattice. However, it should be noted that an excessive amount of flux may produce counterproductive effects, such as leading to the precipitation of dopant ions in the form of oxides, which may weaken the luminescence intensity.

### 4.2 Microwave‐Assisted Reaction

Although the phosphors prepared by the high‐temperature solid‐state reaction method have the advantages of high crystallinity and high doping efficiency, the preparation process requires high temperature and long calcination time, resulting in high energy consumption cost, which has long been a constraint to their practical application.^[^ ^150^, ^151^ ^]^ In recent years, the microwave‐assisted method has gradually become a research hotspot because of its preparation of inorganic luminescent materials with excellent uniformity and flexible morphology control.^[^ ^148^, ^153^, ^154^ ^]^ The core principle of this technology is: through the interaction of electromagnetic radiation and the reaction material, to realize the rapid heating of the raw material in the local area, thus effectively reducing the temperature gradient in the system and significantly shorten the reaction time, and ultimately realize the efficient preparation of luminescent materials.

As shown in Figure 13b, the sample preparation process is as follows: firstly, the homogeneously mixed reaction materials are transferred to an alumina crucible with a capacity of 3–10 mL, which is then placed in a larger crucible (15–30 mL) with a lid to form a double‐crucible structure; the crucible system is steadily placed on the base of the microwave oven (usually powdered activated carbon, which effectively absorbs microwaves up to 2.45 GHz under the condition of room temperature); after confirming that the heat insulation module is in place, the reaction precursor is placed in a microwave environment with different powers and heated for a specific period of time; after the reaction is completed and naturally cooled down, the target phosphor sample can be obtained.

It is worth noting that when preparing doped AZnOS samples, if a reducing atmosphere needs to be introduced, an appropriate amount of carbon powder can be added at the gap of the double crucible, which is due to the fact that carbon can generate CO through incomplete combustion at high temperature. For example, when preparing Eu^2+^ doped phosphors using Eu_2_O_3_ as the dopant precursor, the conversion of Eu^3+^ to Eu^2+^ needs to be realized in a CO gas. Although this process demonstrates the high efficiency and high yield advantages of microwave‐assisted reaction, its inherent limitations should not be overlooked. The use of different raw materials during preparation affects microwave heating efficiency, necessitating precise control of synthesis parameters to optimize final product performance.^[^ ^148^ ^]^ Furthermore, employing carbon powder as a reducing agent may contaminate raw materials, while insufficient microwave power can lead to incomplete reactions.^[^ ^153^ ^]^ Consequently, significant variations in synthesis steps and conditions compromise the reproducibility of the preparation process. Coupled with the high cost of microwave equipment and other drawbacks, careful consideration is required when selecting microwave‐assisted methods for sample preparation.

### 4.3 Multistep‐Assisted Reaction

The solid‐state reaction method has long dominated the phosphor synthesis field as a traditional technique for the preparation of inorganic luminescent materials. However, the samples produced by this method usually have problems such as large particle size and irregular size distribution, and need to be processed by secondary machinery in order to obtain fine‐grained samples that meet the application requirements. In recent years, liquid‐phase synthesis has attracted a lot of attention from the research community due to its unique advantages in the preparation of nanoscale phosphors, especially in the cutting‐edge fields of biomedical imaging.^[^ ^83^ ^]^ In this context, a multistep‐assisted synthesis strategy has emerged, which successfully realizes the customized preparation of phosphors with different specifications by integrating the high crystallinity property of the solid‐state method with the small particle size control capability of the liquid‐phase method.

Take the two‐step synthesis method proposed by Liu et al. as an example: uniform ZnS: Mn^2+^ nanoparticles were first synthesized by a hydrothermal method, and then a Ca(OH)_2_ shell layer was encapsulated on the surface to form a core–shell structural template through a precipitation reaction.^[^ ^155^ ^]^ The template was subjected to high‐temperature sintering at 800 °C under Ar atmosphere, and finally transformed into CaZnOS: Mn^2+^. To optimize the material properties, the research team further employed oleic acid to hydrophobically modify the surface of the product and used polydimethylsiloxane (PDMS) as the matrix to prepare the composites (Figure 13c). Ge et al. also developed a co‐precipitation‐assisted synthetic route for the CaZnOS: Tm^3+^/Yb^3+^ preparation: a combination of the ZnS precursor was mixed with Ca(NO_3_)_2_ solution, dopant ion solution was added dropwise, and the co‐precipitation was induced by adjusting the pH. via NaOH, the resulting precursor was annealed at 1100 °C for 3 h under N_2_ protection to finally obtain the target product with intact crystallization.^[^ ^156^ ^]^

This kind of multistep‐assisted synthesis strategy enables step‐by‐step regulation, achieving precise control of the particle size distribution and providing a new paradigm for material design across diverse applications. Although this method preserves the morphology of the precursor, the preparation steps are cumbersome and difficult to reproduce. Taking Liu et al.'s method as an example, if the uniformity of the Ca(OH)_2_ shell coating on the ZnS: Mn^2+^ surface cannot be guaranteed, the luminescence efficiency of the resulting CaZnOS based phosphors cannot be assured.^[^ ^155^ ^]^ More critically, this approach ultimately requires high‐temperature annealing to ensure crystallinity. From the perspectives of operational efficiency and economic viability, this method is not recommended unless extremely stringent requirements exist for sample morphology and size. Instead, direct preparation via high‐temperature solid‐state reaction is preferable.

### 4.4 Molten‐Salt Shielding Method

As mentioned above, current methods for synthesizing AZnOS rely primarily on solid‐state approaches, which typically result in agglomerated product particles. Despite recent improvements in these solid‐state methods, precise control of the product morphology remains challenging. To address these issues, our research team proposes a novel synthesis method based on a molten‐salt shielding strategy, which effectively solves the particle agglomeration challenges of traditional solid‐state methods by encapsulating solid reactants in a molten‐salt matrix liquefied at high temperatures (Figure 13d).^[^ ^29^, ^149^, ^157^ ^]^ The method achieves physical isolation of the reactants and atmosphere regulation through the molten‐salt medium, which significantly optimizes the dispersion properties of the products while maintaining high crystalline quality.^[^ ^158^ ^]^ Taking the synthesis of CaZnOS: Mn^2+^ as an example, the specific process is as follows: first, the precursor material was mechanically mixed with a salt, i.e., NaCl, at a mass ratio of 1:5, and then transferred to an alumina crucible to form a reaction unit. The reaction was held at 1000 °C for 4 h in ambient atmosphere, during which the intermediate annealing process was completed. At the end of the reaction, the residual salts were removed by washing with DI water several times, and the target product was obtained after drying. The innovativeness of this method is reflected in the following aspects: by selecting NaCl as the medium, whose melting point (801 °C) is significantly lower than the reaction temperature (1000 °C), a continuous liquid‐phase protective encapsulation layer forms during heating. This layer also constructs a physical isolation barrier to effectively inhibit abnormal fusion between particles. The experimental results show that the PL quantum efficiency of CaZnOS: Mn^2+^ prepared by this method reaches 60–80%, and the products present regular flake‐like morphology, and their particle dispersion indexes are reduced by more than 50% compared with those of the traditional high‐temperature solid‐state method.^[^ ^149^ ^]^

It is worth emphasizing that the selection of the molten‐salt medium needs to satisfy two core conditions: first, a melting point lower than the reaction temperature to ensure sufficient liquefaction; second, chemical inertness to avoid participation in the target reaction.^[^ ^157^ ^]^ The molten‐salt shielding method established in this study provides a new idea for the morphology tuning of AZnOS‐based phosphors, enabling precise control of the microstructure while maintaining a high crystallinity in the prepared products. However, it is important to note that the addition of salt significantly reduces the required reaction conditions. Consequently, excessively high reaction temperatures or prolonged calcination times may cause decomposition of the synthesized AZnOS, thereby compromising the purity of the final product. Additionally, residual salts must be washed off with DI water during sample collection, which may also affect product quality to some extent.

## 5 Related applications

AZnOS have garnered significant attention due to their exceptional optical properties, which unlock diverse applications in displays, smart materials, anti‐counterfeiting technologies, and security systems.^[^ ^23^, ^25^, ^31^ ^]^ These phosphors exhibit transformative potential for enhancing material reliability, fueling smart technology innovation, and reducing energy consumption. Their cost‐effective and eco‐friendly synthesis processes further accelerate adoption, addressing escalating demands for advanced visual experiences while promoting sustainability and technological progress. Multimode luminescent materials are pivotal for flexible optoelectronics, information encryption, and structural health monitoring, as they enable contact‐free sensing and dynamic real‐time visualization.^[^ ^159^ ^]^ However, the advancement of optical sensing technologies remains constrained by the scarcity of high‐performance luminescent platforms that can simultaneously achieve sensitivity, stability, and scalability. AZnOS emerges as a promising candidate, demonstrating exceptional versatility as a multimode luminescent system. Its applications span stress/strain sensing, high‐resolution bioimaging, and adaptive intelligent lighting.^[^ ^160^, ^161^, ^162^ ^]^ Notably, the ML phenomenon in AZnOS, a passive, energy‐autonomous process triggered by mechanical stress, aligns with modern imperatives for low‐carbon technologies, offering energy‐efficient solutions for environmental conservation and sustainable development. Through strategic heterojunction engineering and ion‐doping strategies, AZnOS achieves multifunctional capabilities in energy harvesting, multimodal sensing (e.g., strain‐temperature‐pressure mapping), secure optical encryption, and advanced anti‐counterfeiting. The subsequent sections will elaborate on specific implementations of AZnOS in optics‐based applications, with details provided in the following subsections.

### 5.1 Mechanical‐Photonic Conversion and Sensor

The mechanical‐photonic conversion properties of AZnOS (especially CaZnOS and SrZnOS) enable their ML response range to be extended to very low pressure levels by a low activation pressure threshold.^[^ ^25^, ^159^ ^]^ Such excellent ML properties endow the AZnOS with broad application prospects in the fields of stress visualization and dynamic pressure sensing, which not only deepen the understanding of the mechanism of mechanical‐photonic conversion, but also improves the luminescence performance of ML materials, which is expected to provide important guidance for the development of high‐performance ML materials.

**Figure 14:**
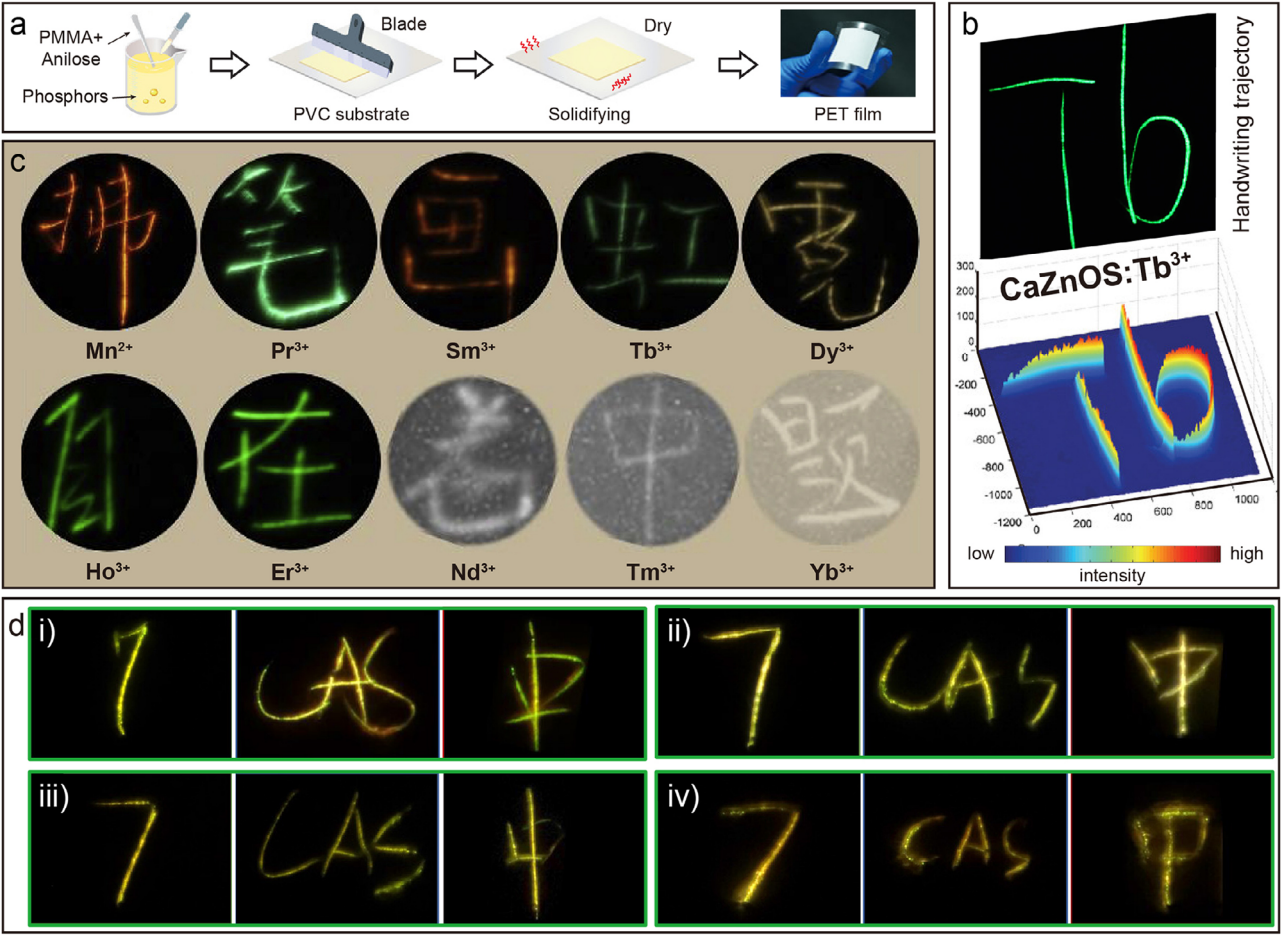
a) Schematic diagram of the preparation procedure of ML composite film. Reproduced with permission.^[^ ^163^ ^]^ Copyright 2022, American Chemical Society. b) Visualization of handwriting trajectory through long‐time exposure ML imaging of CaZnOS: Tb^2+^ (top) and the distribution of relative ML intensity extracted from the image according to the gray scale value (bottom). Reproduced with permission.^[^ ^35^ ^]^ Copyright 2019, Wiley‐VCH. c) Photographic images recording the motion trails of a pen on the surface of the ML cylinders. The exposure time for each photo was 2 s. Reproduced with permission.^[^ ^53^ ^]^ Copyright 2020, Elsevier. d) Signatures of four different people were captured (i–iv) by a mobile phone in long exposure mode, where the stress distribution was distinguished by color changes visible to the naked eye in the dark. Reproduced with permission.^[^ ^121^ ^]^ Copyright 2022, Wiley‐VCH.

In order to quantitatively assess ML performance and validate the ability of AZnOS to image stress distributions, pressure mapping of personalized handwriting is the most intuitive application example. It is often necessary to encapsulate the phosphor in an organic polymer matrix for testing, for example, a mixture of poly(methyl methacrylate) (PMMA) and anisole is firstly selected to be mixed with the AZnOS based phosphor to form a homogeneous paste; subsequently, a transparent poly(vinyl chloride) (PVC) film with a thickness of 1 mm is used as a flexible substrate, and the paste is uniformly scraped on the surface of the substrate; finally, the compound film is dried and cured to form a composite film for subsequent ML characterization (**Figure** **14**a).^[^ ^163^ ^]^ In addition to the above matrices, elastic organic polymers such as epoxy resin, PDMS, silicone rubber, and polyethylene terephthalate (PET) are also commonly used in the preparation of ML composites, and these polymers possess the characteristics of high dispersibility, optical transparency, and compatibility, which can minimize the influence of external pressure on the ML properties of the materials. As a result, when mechanical stress is applied to the composite film, the AZnOS based phosphor can release photons rapidly and form a luminescent trace that is highly consistent with the trajectory of the mechanical stress. Notably, the ML intensity of AZnOS is positively correlated with the applied force. Taking the study of Wang's team as an example, it achieved the visualization of the handwriting traces by prolonging the ML imaging exposure time of CaZnOS: Tb^3+^, and further extracted the image gray values to analyze the relative ML intensity variations at different points on the traces (Figure 14b).^[^ ^35^ ^]^ In addition, by single doping of Ln or other luminescent ions, AZnOS can be made to realize tunable multi‐band ML, and the emission wavelength range can be extended from the VIS to the NIR region. For example, Xie et al. wrote Chinese characters with a ballpoint pen on the surface of SrZnOS: Ln^3+^ or Mn^2+^ films, and their motion trajectories could be successfully recorded by an ordinary camera or a NIR camera in a dark environment (Figure 14c).^[^ ^53^ ^]^ More interestingly, a stress‐sensing strategy based on ratiometric dual emission can achieve stress‐sensitive ML color changes in Ln/TM co‐doped AZnOS. As shown by Wang's team, the ML color change of CaZnOS: Tb^3+^/Mn^2+^ can reflect the difference in the pressure exerted on each stroke during the writing process, and thus determine the writing habits of individuals (Figure 14d).^[^ ^121^ ^]^

**Figure 15:**
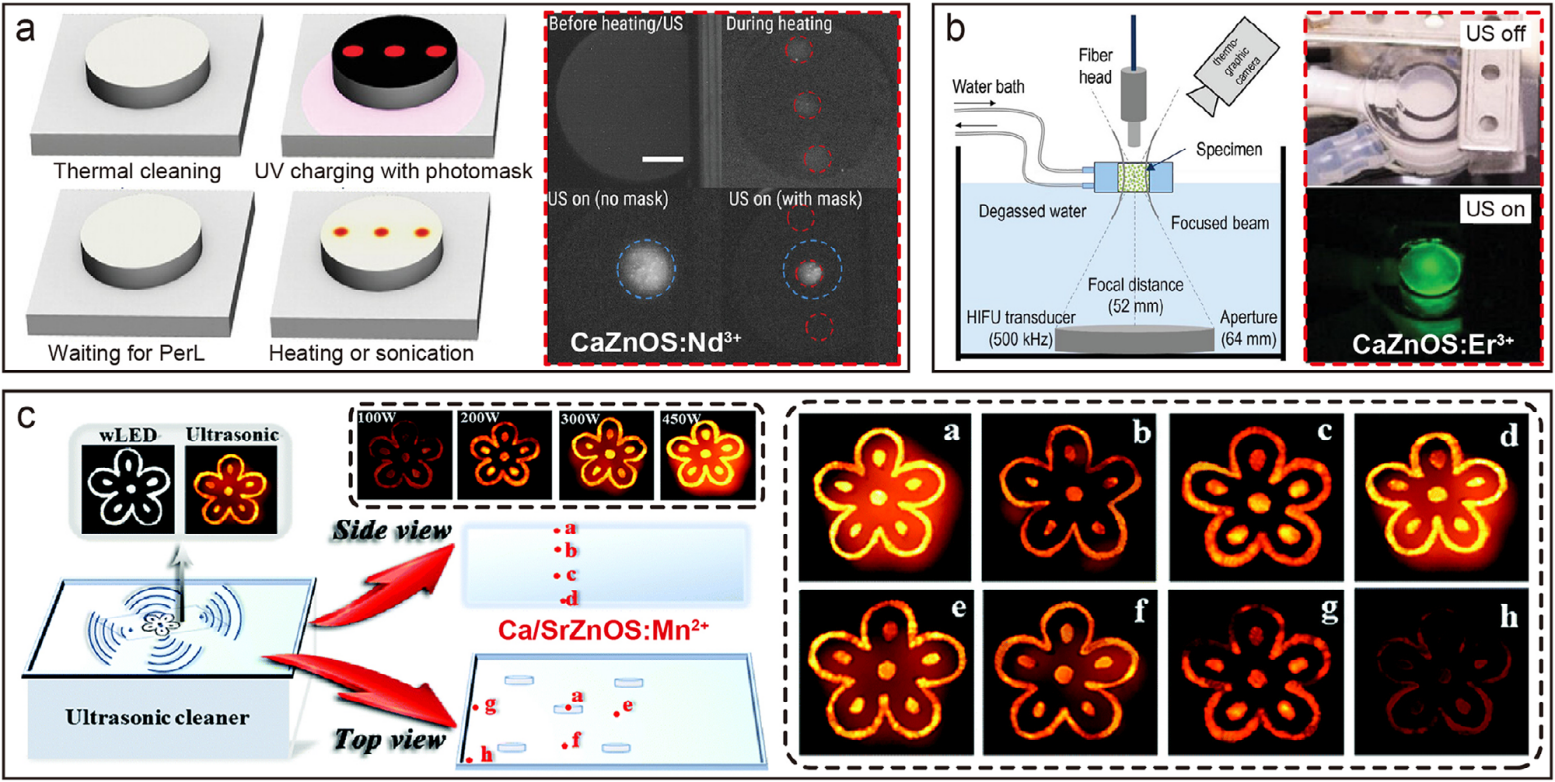
a) Schematic of an energy storage and information read‐out procedure (left) and corresponding camera images taken on CaZnOS: Nd^3+^ in an epoxy resin (right). Reproduced with permission.^[^ ^152^ ^]^ Copyright 2023, Wiley‐VCH. b) Schematic of the experimental set‐up used for quantitative HIFU‐induced ML (left), and the ML photo of CaZnOS: Er^3+^ placed in a peripheral ring cuvette with recirculating water and its dark environment with US on (right). Reproduced with permission.^[^ ^46^ ^]^ Copyright 2022, Wiley‐VCH. c) Schematic of distribution position in the US cleaner, with insets showing photos of Ca/SrZnOS: Mn^2+^ at different powers (left) and at different locations in the US cleaner (right). Reproduced with permission.^[^ ^128^ ^]^ Copyright 2021, The Royal Society of Chemistry.

In addition to applying mechanical stress directly to the complex film, ML imaging using ultrasonic (US) excitation of AZnOS is also a preferred option for in vivo imaging tasks. Compared to other mechanical stimulation modalities, US excitation avoids large displacements and the risk of host damage, and can penetrate aqueous media, biological tissues, or other materials without direct contact.^[^ ^164^, ^165^ ^]^ For example, Wondraczek's team verified the PersL performance of CaZnOS: Nd^3+^ and the synchronized response of US‐excited ML to temperature, light, and acoustic loads (**Figure** **15**a), enabled multimodal data storage and readout, and confirmed the remote activation capability of CaZnOS in water and biological tissues.^[^ ^152^ ^]^ In another study, they constructed a high‐intensity focused ultrasound (HIFU) experimental setup with a power of 40 W (293 K) for quantitative ML testing, and successfully realized the visible ML of CaZnOS: Er^3+^ (Figure 15b), which fully demonstrated the potential of US‐induced ML in the field of optical information storage and optical temperature measurement.^[^ ^46^ ^]^ Furthermore, a common laboratory US cleaner can also be used as an excitation source for AZnOS to monitor and visualize the US intensity. For example, Zhang's team used “cherry blossom‐like” films prepared with Ca/SrZnOS: Mn^2+^ and observed significant brightness changes under different power and US intensity (Figure 15c).^[^ ^128^ ^]^ At present, most phosphors capable of achieving long afterglow through ultrasonic activation belong to the PersL category, and the duration of afterglow is directly correlated with defect depth.^[^ ^47^, ^131^ ^]^ Therefore, phosphors with deeper energy level defects are typically selected as host materials to ensure prolonged afterglow. Compared to commercial phosphors such as SrAl_2_O_4_: Eu^2+^/Dy^3+^ and Y_2_O_2_S: Eu^3+^/Mg^2+^/Ti^4+^,^[^ ^166^, ^167^ ^]^ which exhibit afterglow lasting hours or even days following UV excitation, AZnOS‐based PersL phosphors struggle to match this performance. Therefore, strategically modifying AZnOS and introducing deep defects is a key approach to realizing US‐excited ML applications.

**Figure 16:**
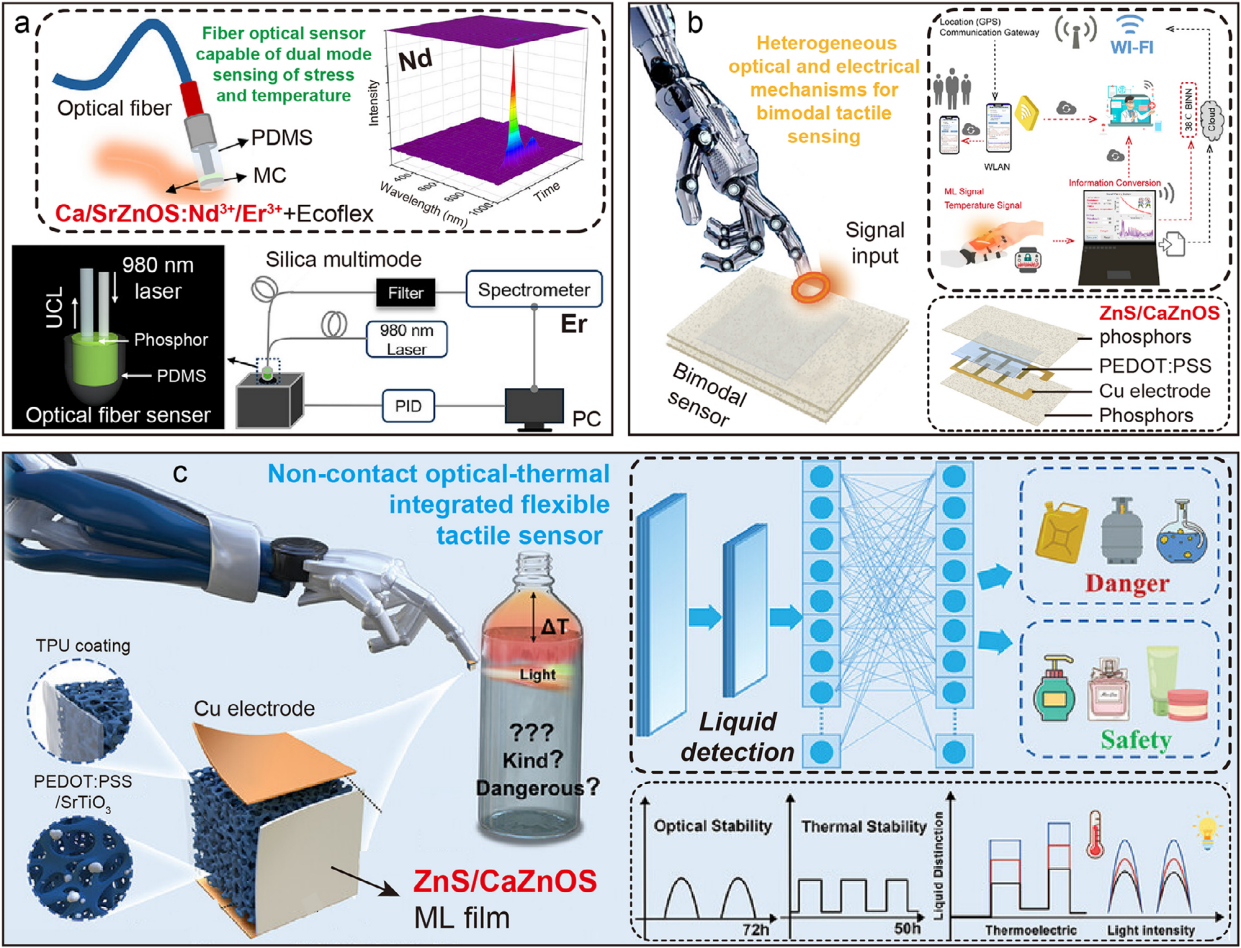
a) A probe‐like structure made of Ca/SrZnOS: Nd^3+^/Er^3+^and fiber optics, and the ML spectrograms corresponding to probe strokes (top), and the self‐constructed optical fiber temperature detection system (bottom). PID is proportional‐integral‐derivative. Reproduced with permission.^[^ ^119^ ^]^ Copyright 2025, Wiley‐VCH. b) Conceptual mechanism of ZnS/CaZnOS heterojunction sensing combining ML effects and thermal resistance for bimodal tactile sensing. The insets show a schematic of wireless data transmission for remote interaction (top) and the structure of a bimodal tactile sensor (bottom). Reproduced with permission.^[^ ^172^ ^]^ Copyright 2022, American Chemical Society. c) The schematic diagram of the optical–thermal integrated flexible tactile sensor for high‐fine recognition of liquid properties under non‐contacting mode. Reproduced with permission.^[^ ^173^ ^]^ Copyright 2024, Wiley‐VCH.

The multiple luminescence modes of AZnOS (e.g., PL, ML, and UCL, etc.) open up new avenues for innovative sensing applications, especially in optical sensors. Currently, with the development of technology, single‐function phosphors with non‐tunable emission wavelengths (e.g., ZnS: Cu^+^/Cl^−^) no longer meet the demands of complex application scenarios and the precision required for information transmission.^[^ ^168^ ^]^ Therefore, methods to realize the coupling of various luminescence modes in AZnOS based phosphors present significant research demand for stable luminescence.^[^ ^169^, ^170^, ^171^ ^]^ Based on this, Chen et al. developed a dual‐mode sensor device capable of stress and temperature sensing (**Figure** **16**a), which realized efficient NIR ML (from Nd^3+^) and UCL (from Er^3+^) excited by a 980 nm laser owing to the excellent luminescence properties and temperature stability of Ca/SrZnOS: Nd^3+^/Er^3+^.^[^ ^119^ ^]^ This research plays an important role in the development of more accurate and sensitive multimodal sensors for other researchers in the field; however, intuitive and interference‐free reading of multiple tactile signals without involving complex algorithms and computations remains a serious challenge in the field of tactile sensors. Pan and colleagues proposed a bimodal sensor for temperature and pressure without signal fusion (Figure 16b), which was realized by the ML of ZnS/CaZnOS: Mn^2+^ for visual recognition and by the thermoresistance effect of PEDOT: PSS for temperature sensing.^[^ ^172^ ^]^ Finally, a dual‐mode sensing application with temperature sensitivity of −0.6% °C^−1^ and 2 N minimum force detection was realized by tactile sensing. Although some research progress has been made in the field of bimodal sensors for user interaction applications, a major challenge in this field is the inability to accurately recognize liquids because it is not possible for these flexible tactile sensors to apply force directly to liquids. To overcome these challenges, Li et al. proposed a novel optical–thermal multifunctional flexible sensor strategy for precise recognition of liquids.^[^ ^173^ ^]^ They integrated PEDOT: PSS/SrTiO_3_ thermoelectric foam and ML film made of ZnS/CaZnOS: Mn^2+^ into a device platform, and achieved precise recognition accuracy of ≈95% and 97.5% with the help of a multimodal sensing data fusion algorithm based on thermal and optical outputs, which can distinguish between different kinds of pure solvents and turbid suspensions, respectively. The above works not only promote the research avenues for future innovations in flexible electronic devices, but also have great significance for the design and application of next‐generation smart devices.^[^ ^174^, ^175^, ^176^, ^177^, ^178^ ^]^

### 5.2 Anti‐Counterfeiting and Thermometry

AZnOS‐based phosphors are capable of generating highly complex and difficult‐to‐copy coded information due to their excellent optical properties, which can effectively enhance the anti‐counterfeiting level of products.^[^ ^179^ ^]^ By selecting different dopant ions and their combinations, a wide range of security markings can be designed on demand. Such markings can be triggered by specific excitation methods (e.g., UV, mechanical stress, or temperature), which facilitates fast and accurate identification. Notably, AZnOS based phosphor can maintain stable luminescence performance in a wide range of temperature and long‐term illumination conditions, Compared to other inorganic luminescent materials such as sulfides (like CaS: Eu^2+^/Dy^3+^),^[^ ^180^ ^]^ fluorides (like NaYF_4_: Ln^3+^@NaYF_4_),^[^ ^181^ ^]^ and metal halides (like Cs_3_Cd_2_Cl_7_: Sb^3+^/Mn^2+^),^[^ ^182^ ^]^ AZnOS demonstrates stronger stability, which is crucial to ensure the longevity and reliability of anti‐counterfeiting marks. In addition, with the popularization of the concept of environmental protection, AZnOS‐based phosphor has been proven to be environmentally friendly, aligned with the trend of green development.

**Figure 17:**
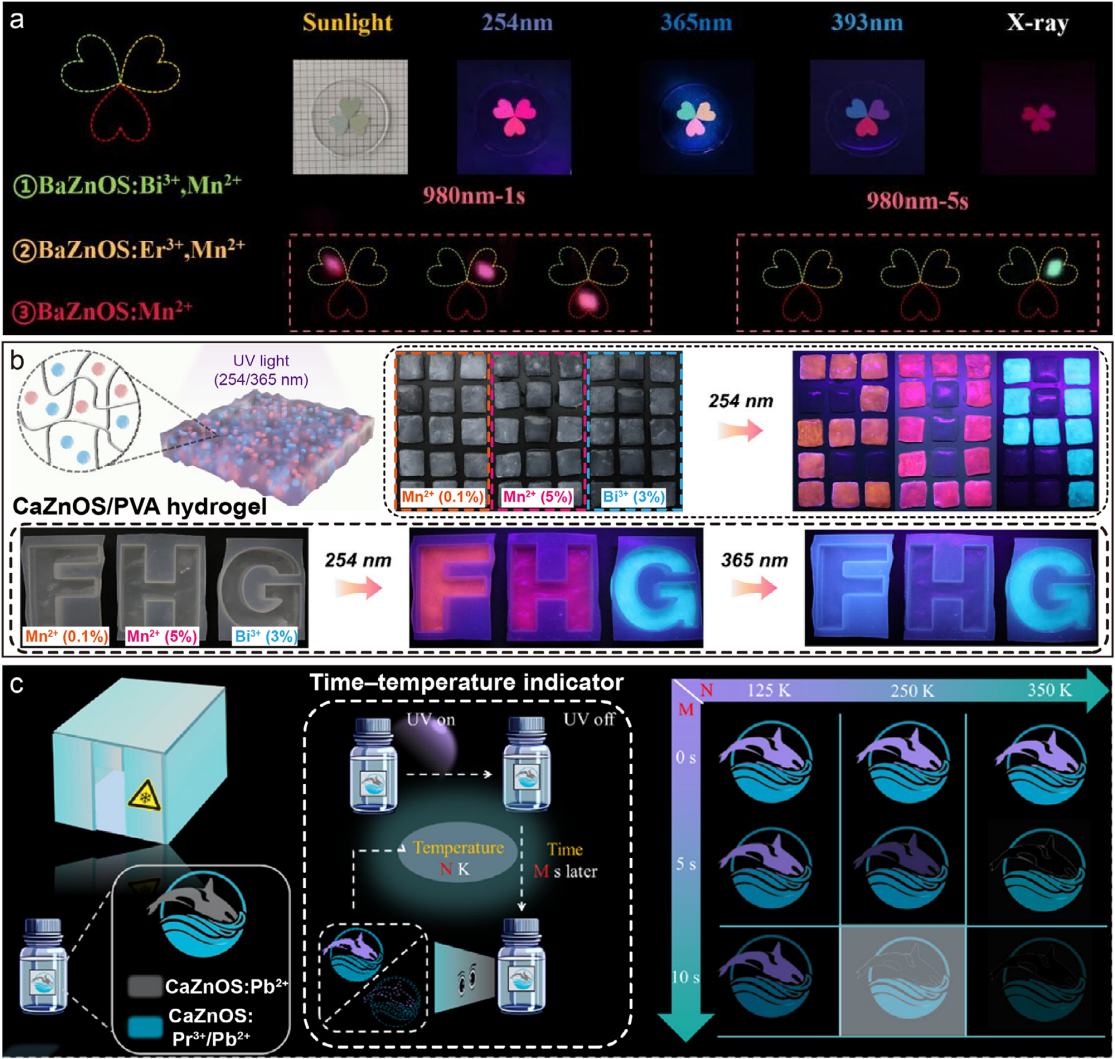
a) PDMS composited BaZnOS: Bi^3+^/Mn^2+^, BaZnOS: Er^3+^/Mn^2+^, and BaZnOS: Mn^2+^ films under different excitation sources. Reproduced with permission.^[^ ^120^ ^]^ Copyright 2022, Elsevier. b) Schematic of the multicolor fluorescent hydrogels with patterns of different numbers and letters treated with CaZnOS: Bi^3+^ and CaZnOS: Mn^2+^ under visible and UV irradiation. Reproduced with permission.^[^ ^183^ ^]^ Copyright 2025, American Chemical Society. c) Visualizing the time‐dimensional response of cryogenic temperature detection in refrigeration applications, including a cryogenic detection logo used in cold storage (left), the mechanism by which the logo works (mid), and an image of the cryogenic time‐temperature response of CaZnOS: Pr^3+^/Pb^2+^ (right). Reproduced with permission.^[^ ^123^ ^]^ Copyright 2024, American Chemical Society.

To meet the advanced anti‐counterfeiting needs of spatio‐temporal differentiation, Yu et al. composited three samples of BaZnOS: Bi^3+^/Mn^2+^, BaZnOS: Er^3+^/Mn^2+^, and BaZnOS: Mn^2+^ with PDMS composites, flexible films with “cloverleaf” morphology were prepared (**Figure** **17**a).^[^ ^120^ ^]^ Under the irradiation of different excitation wavelengths, different regions of the film can show color differences, which, combined with the dual response characteristics of stress and temperature, significantly improve the security of anti‐counterfeiting and expand the boundary of the practical application of the fluorescent anti‐counterfeiting technology. Based on the complex combination of tunable emission colors, AZnOS‐based phosphor can achieve higher density information storage, and then doped into the polymer gel matrix prepared by the fluorescent hydrogel, both gel properties and luminescence. For example, Jiang's team doped different concentrations of CaZnOS: Bi^3+^ and CaZnOS: Mn^2+^ into poly(vinyl alcohol) (PVA) hydrogels by US emulsification and successfully constructed a variety of luminescent letter patterns.^[^ ^183^ ^]^ By switching between 254 and 365 nm excitation sources, the letter colors can be dynamically adjusted from orange to blue (Figure 17b), which greatly improves the information capacity. In addition to the diversity of luminescent colors, temperature is also a key parameter to regulate the luminescent properties of AZnOS. Among them, the optical temperature measurement technique based on fluorescence intensity ratio exhibits high sensitivity and color resolution in the low‐temperature region (<300 K), which provides an effective means to visualize temperature information. Huang et al. developed a time‐responsive cryogenic detection strategy based on the dynamic optical signals of CaZnOS: Pr^3+^/Pb^2+^ (Figure 17c), and their flexible time‐temperature indicating tags prepared by mixing the samples with PDMS can be used in real‐time by the process of carrier release from the ultra‐shallow traps to visualize the storage duration and temperature information of the product during transportation.^[^ ^123^ ^]^ This study provides a convenient, efficient, and visualized solution for cryogenic monitoring in the fields of cold chain transportation, medical cold storage, and cryogenic bioengineering.

**Figure 18:**
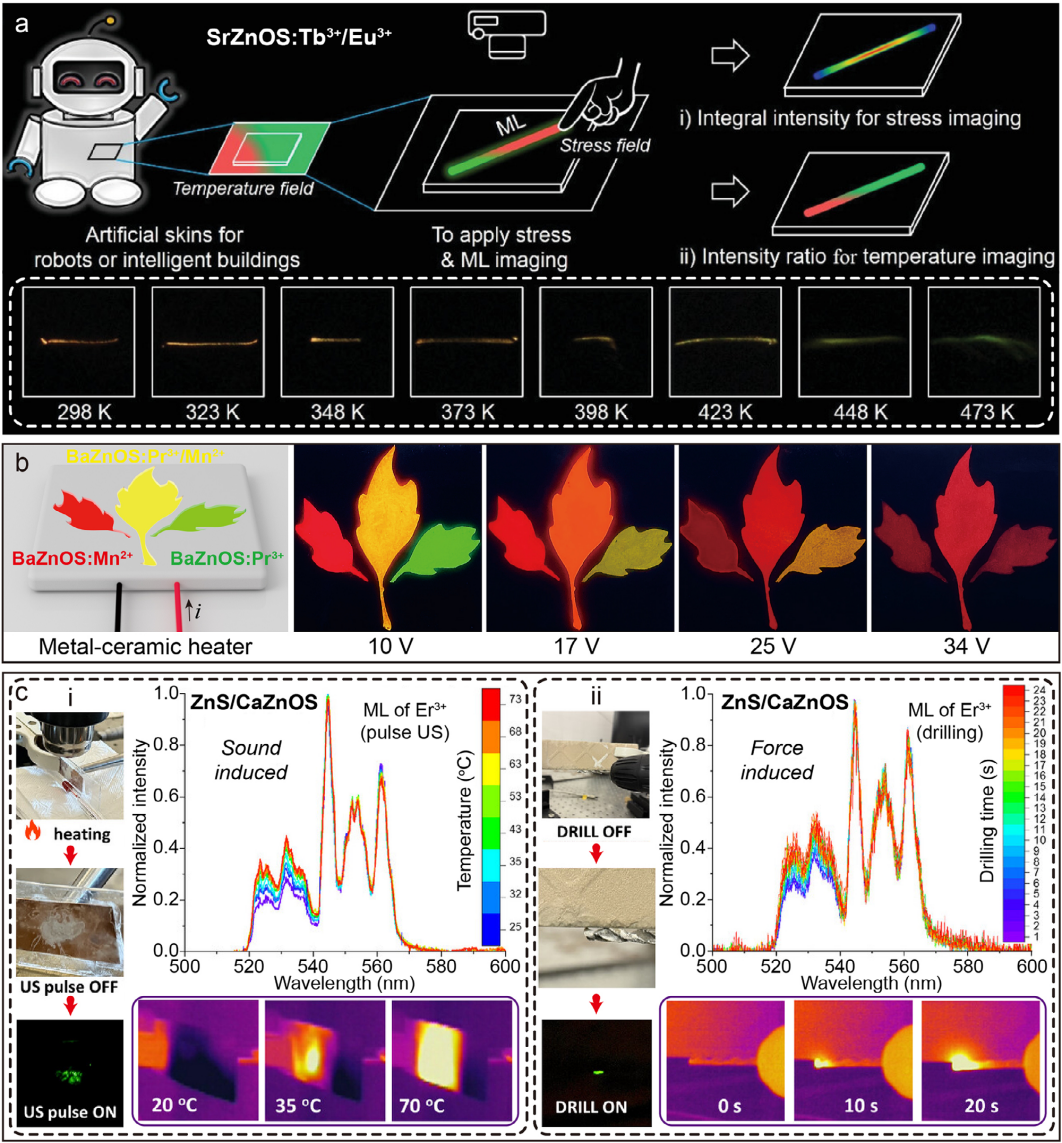
a) Schematic of the imaging system and the stress‐temperature imaging principle (top), and photos of the ML film being scraped at different temperatures (bottom). The exposure time of the image sensor is 0.5 s. Reproduced with permission.^[^ ^57^ ^]^ Copyright 2021, Wiley‐VCH. b) Schematic of the electrochromic device consisting of a metal–ceramic heater substrate with BaZnOS: Mn^2+^, BaZnOS: Pr^3+^/Mn^2+^, and BaZnOS: Pr^3+^ film (left). And the temperature‐dependent photos of the maple leaf pattern under UV excitation (305 nm) at different applied voltages (right). Reproduced with permission.^[^ ^58^ ^]^ Copyright 2022, Wiley‐VCH. c) ZnS/CaZnOS: Er^3+^ is used for (i) an external heating system for remote temperature detection, and (ii) luminescence thermometry induced by localized heating through drilling. They contain self‐made test apparatus, luminescence spectra captured at different temperatures or times, and thermal imaging pictures. Reproduced with permission.^[^ ^137^ ^]^ Copyright 2025, Wiley‐VCH.

The great potential of luminescence temperature measurement technology has driven the intensive study of different doped ion combinations with multimode emission properties, among which co‐doped systems containing two types of Ln^3+^ (e.g., Tb^3+^/Eu^3+^) are of particular attention. Temperature sensing based on the electron distribution among different excited states can be realized by various excitation methods, such as UCL and DCL, but both of these methods require an external light source for pumping or charging.^[^ ^137^, ^156^, ^184^, ^185^ ^]^ Therefore, the development of self‐supplied luminescence temperature measurement technology without light source excitation is expected to bring a revolutionary breakthrough in the field of temperature sensing. Xie et al. constructed a stress‐temperature dual‐modal imaging scheme based on the SrZnOS: Tb^3+^/Eu^3+^ (**Figure** **18**a).^[^ ^57^ ^]^ This scheme achieves temperature detection with a *S_r_*_max_ of 0.877% K^−1^ in the 298–473 K region by integrating the integral luminescence intensity of the stress response with the temperature‐dependent fluorescence intensity change. This sensing system remotely visualizes the spatial and temporal distributions of stress and temperature without the need for photoexcitation, providing a simple, efficient, and reproducible solution for multiparameter synergistic sensing. Moreover, the co‐doped luminescent system of Ln and TM exhibits remarkable temperature‐dependent properties. In particular, the efficient energy transfer between the two doped ions varies with temperature, endowing the AZnOS‐based phosphor with excellent dynamic temperature sensing performance. For example, Wang's team integrated three samples, BaZnOS: Pr^3+^/Mn^2+^, BaZnOS: Pr^3+^, and BaZnOS: Mn^2+^, into an electrochromic device and achieved a precise temperature response through voltage modulation (Figure 18b).^[^ ^58^ ^]^ With increasing temperature, BaZnOS: Pr^3+^/Mn^2+^ (*S_r_*_max_ = 8.58% K^−1^) and BaZnOS: Pr^3+^ (*S_r_*_max_ = 4.93% K^−1^) respectively, show significant color changes from yellow/green to red, providing an intuitive visual signature for temperature modulation. In addition to the dual‐doped ion thermometers described above, Zheng et al. recently employed Er^3+^‐doped ZnS/CaZnOS heterojunctions to achieve remote temperature sensing via an external heating system, and localized object heating through mechanical friction (i.e., drilling) for luminescence‐based temperature measurement.^[^ ^137^ ^]^ As shown in Figure 18c, the combination of sound/force‐induced ML and luminescence thermometry for the ZnS/CaZnOS heterojunction demonstrates outstanding performance, with the thermalization phenomenon attributable to the Er^3+^ emission bands clearly observable. These studies not only expand the application scenarios of luminescence temperature measurement technology through the ion doping strategy and device integration innovation, but also provide a new technological path for multimodal sensing and dynamic temperature modulation, which has an important application prospect in the fields of industrial temperature control and biomedicine.

### 5.3 Display and Lighting

**Figure 19:**
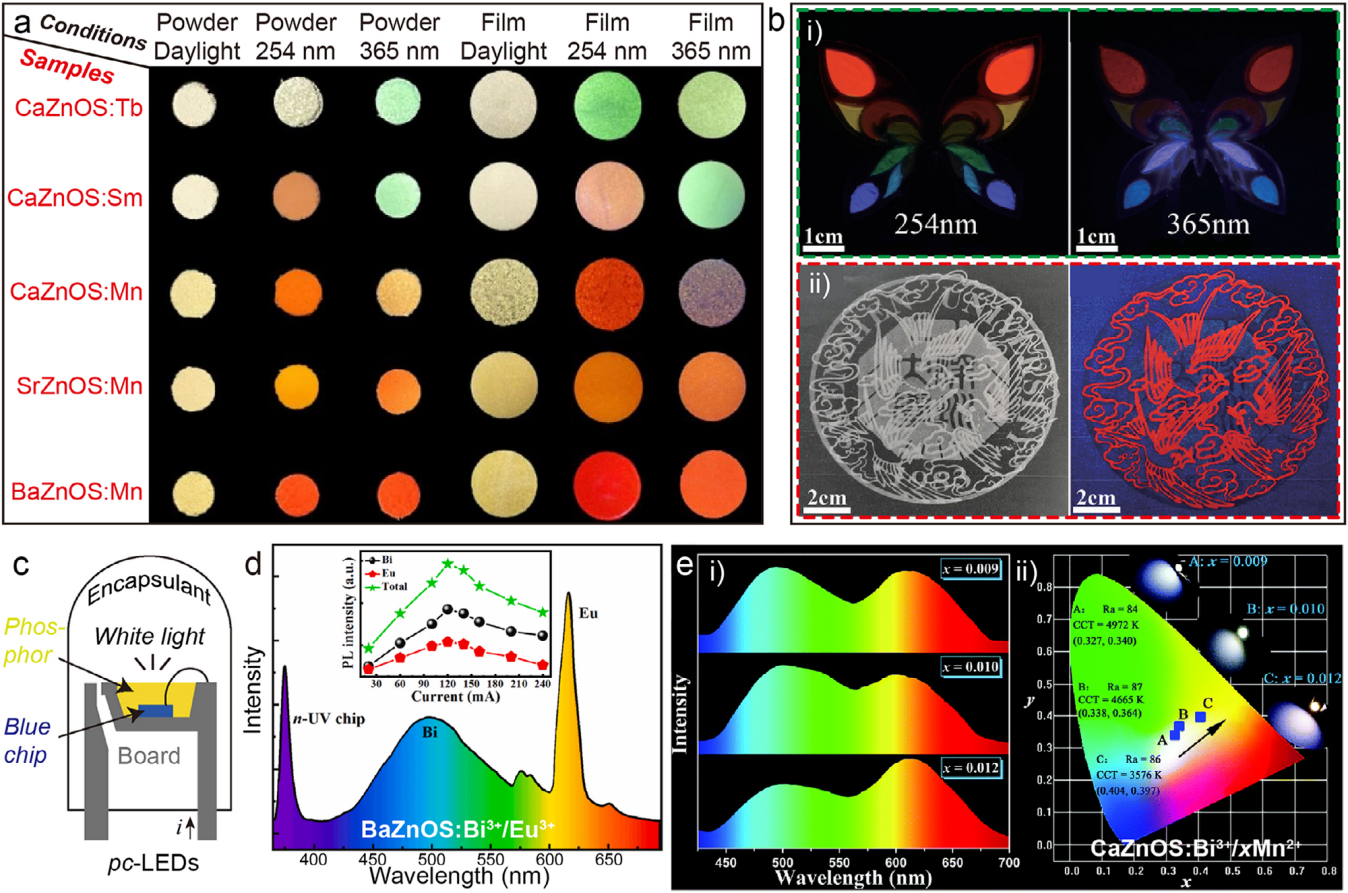
a) Photographs of AZnOS‐based phosphors or film under daylight and UV light (254 and 365 nm). Reproduced with permission.^[^ ^154^ ^]^ Copyright 2024, Elsevier. b) i: The butterfly molds filled with different samples were irradiated with 254 and 365 nm UV lights, respectively; ii: The patterns drawn using the printing technique were irradiated under daylight and 254 nm UV light, respectively. Reproduced with permission.^[^ ^149^ ^]^ Copyright 2022, Wiley‐VCH. c) Structure of white *pc*‐LEDs. Reproduced with permission.^[^ ^186^ ^]^ Copyright 2019, Wiley‐VCH. d) Emission spectra of the *pc*‐LEDs system with BaZnOS: Eu^3+^/Bi^3+^ encapsulated into a cap that is placed on top of a *n*‐UV chip, the inset shows the corresponding emission intensities of Bi^3+^, Eu^3+^, and total when the driven current increases from 20 to 240 mA. Reproduced with permission.^[^ ^54^ ^]^ Copyright 2023, Elsevier. e) i: EL spectra of the *pc*‐LEDs fabricated with the CaZnOS: Bi^3+^/*x*Mn^2+^ (*x*= 0.9, 1.0, and 1.2%) and a 365 nm UV chip; ii: CIE chromaticity diagram corresponding to i. Reproduced with permission.^[^ ^187^ ^]^ Copyright 2021, The Royal Society of Chemistry.

The host absorption band of AZnOS‐based phosphors is located near 300 nm, which can efficiently convert UV or blue light into multicolored VIS (**Figure** **19**a), providing a key material support for the construction of high‐efficiency and low‐energy‐consumption solid‐state lighting systems.^[^ ^154^ ^]^ By choosing different dopant ions, luminescence modulation covering the entire visible spectrum can be realized. For example, by doping CaZnOS with different luminescent ions into home‐made butterfly molds, our team observed rich multicolor luminescence under 254 and 365 nm UV excitation, respectively (Figure 19b i).^[^ ^149^ ^]^ Further, the overlapping patterns of CaZnOS doped with Bi^3+^ and Mn^2+^ prepared by the screen‐printing technique exhibited bright composite luminescent colors in the overlapping regions under 254 nm UV excitation (Figure 19b ii). Such multicolor luminescent properties not only expand the color spectrum of display technologies, but also provide an important technological path for the development of new display technologies such as quantum dots (QDs) displays. Therefore, the application of AZnOS in lighting and display not only promotes the innovation of display technology but also demonstrates significant social benefits in terms of energy saving, emission reduction, and environmental protection. With the continuous optimization of material design and preparation process, the application prospects of inorganic luminescent materials will be broader.

In the field of lighting, LEDs, as a highly efficient electro–optical conversion semiconductor device, have become a dominant lighting source by virtue of their cost‐effectiveness, long life, and low power consumption.^[^ ^118^, ^188^ ^]^ Among them, white light *pc*‐LEDs based on the combination of UV chips and phosphors are the most common realization (Figure 19c).^[^ ^186^ ^]^ Yttrium aluminum garnet (YAG) is widely used in *pc*‐LEDs devices due to its excellent thermal stability and chemical durability.^[^ ^189^ ^]^ For instance, Ce^3+^ within the YAG lattice undergoes efficient 4*f*‐5*d* transitions that generate high‐efficiency yellow emission. When paired with UV chips, YAG: Ce^3+^ produces white light with a high color rendering index and optical efficacy. Inspired by this, researchers are progressively exploring the application potential of AZnOS‐based phosphors in this field. For example, Dai et al. integrated BaZnOS: Eu^3+^/Bi^3+^ into the package lid of a commercial AlInGaN UV chip, which was driven by 120 mA current at 375 nm (UV chip), 491 nm (Bi^3+^), and 614 nm (Eu^3+^) showing overlaid white light emission (Figure 19d).^[^ ^54^ ^]^ Notably, the luminescence intensity of Bi^3+^, Eu^3+^ and the total PL intensity are all linearly enhanced with the increase of the driving current (inset of Figure 19d) until saturation at 120 mA. In addition, Zhang's team used a 365 nm UV chip in combination with CaZnOS: Bi^3+^/Mn^2+^ to achieve color temperature modulation from cool‐white to warm‐white by adjusting the Mn^2+^ doping concentration (0.9, 1.0, and 1.2%) at 3.2 V voltage and 300 mA current (Figure 19e i).^[^ ^187^ ^]^ And the corresponding optical performance of the device is good: the color coordinates are (0.327, 0.340), (0.338, 0.364), and (0.404, 0.397), and the associated color temperatures are 4972, 4655, and 3576 K, respectively (Figure 19e ii). The above study fully verifies that AZnOS possesses high stability and luminescent efficiency in *pc*‐LEDs devices, which lays a solid foundation for their practical application in the lighting field.

### 5.4 Biomedical and Agricultural

NIR light (700–1700 nm) shows unique advantages in the field of biomedical imaging due to its excellent spatial resolution and tissue penetration ability.^[^ ^118^ ^]^ Optical radiation in this wavelength band can effectively reduce the energy attenuation caused by tissue scattering, and also avoid the interference of self‐fluorescence from biological tissues, which provides a technological basis for realizing in situ biomechanical monitoring in vivo.^[^ ^126^, ^152^ ^]^ Based on this physical property, researchers have developed AZnOS‐based phosphors doped with NIR‐emitting Ln, which can generate the ML under mechanical stimuli (e.g., tissue contraction, US action). Notably, the AZnOS possess self‐recovering ML properties and do not require external charging, making them a significant potential for application in biomedical scenes requiring localized light sources, such as optogenetic modulation and photodynamic therapy.

**Figure 20:**
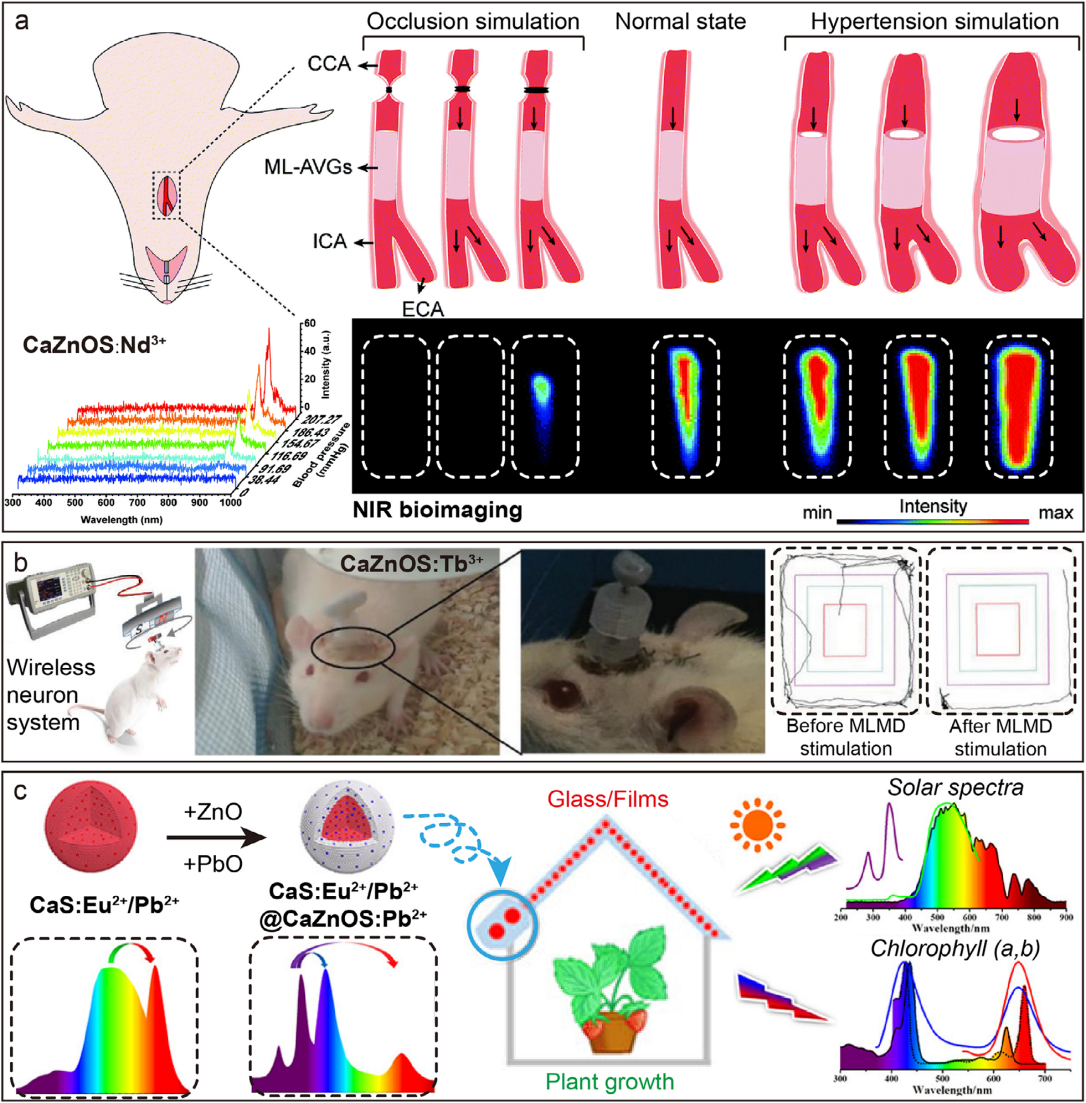
a) Thermal imaging and degree of opening of the common carotid artery, schematic diagrams of normal state and epinephrine injection, and NIR bioimaging of AVG in the corresponding states. Inset shows CaZnOS: Nd^3+^ AVG at different blood pressures in the common carotid artery vascular occlusion and hypertensive model. Reproduced with permission.^[^ ^191^ ^]^ Copyright 2022, The Royal Society of Chemistry. b) Schematic diagram of the homemade MLMD system, photographs of the MLMD device implanted in the brain regions of mice and C1V1 protein expression, and movement trajectories of mice before and after different stimuli. Reproduced with permission.^[^ ^192^ ^]^ Copyright 2021, Wiley‐VCH. c) Preparation mechanism of CaS@CaZnOS core–shell structures and their spectral conversion design for plant growth applications, including solar and excitation spectra, as well *chlorophylls (a,b)* absorption and emission spectra of CaS: Eu^2+^/Pb^2+^@CaZnOS: Pb^2+^. Reproduced with permission.^[^ ^136^ ^]^ Copyright 2021, American Chemical Society.

In the field of biomechanical imaging, NIR ML has demonstrated breakthrough advantages. By implanting NIR ML phosphors into deep tissues (e.g., subcutaneous tissues or the central nervous system), non‐invasive monitoring of the mechanical status can be realized.^[^ ^122^, ^190^ ^]^ Mao's group constructed composite grafts by compositing CaZnOS: Nd^3+^ into artificial vascular grafts (AVG).^[^ ^191^ ^]^ They successfully realized real‐time monitoring of cardiovascular disease models by utilizing the CaZnOS: Nd^3+^ as a biomechanical probe. As shown in **Figure** **20**a, in NIR imaging, the ML signal intensity was positively correlated with the blood pressure gradient in the hypertension model, whereas in the vascular occlusion model it showed a dynamic feature with the degree of vascular recanalization. Spectral analysis further confirmed that the NIR ML intensity showed a linear enhancement trend with increasing blood pressure (inset of Figure 20a), suggesting that the system can accurately reflect the in vivo working environment of the ML‐AVG. In the field of wireless neuromodulation, Chang's team developed CaZnOS: Tb^3+^ based magneto‐luminescent microdevice (MLMD) to construct a remote optogenetic stimulation system.^[^ ^192^ ^]^ The device excites the ML response of CaZnOS: Tb^3+^ through mechanical deformation triggered by an external magnetic field, realizing a contactless photon output. As shown in Figure 20b, when the MLMD system was used for neuromodulation in freely moving mice, a significant reduction in the movement distance of the mice after stimulation was found by maze behavioral analysis. This study demonstrates that the MLMD system can be used as a novel tool for deep nerve stimulation to achieve precise neuromodulation without invasive surgery. Although the above‐mentioned studies confirm the application potential of AZnOS‐based phosphors in non‐invasive bioimaging and biomedical fields, their emission efficiency in the NIR region remains slightly inferior to that in the VIS. It is instructive that prior research indicates SrZnOSe‐based phosphors with the same Ca(Sr)ZnOS structure exhibit high‐performance self‐recovery ML properties within the NIR emission range.^[^ ^26^ ^]^ This is primarily attributed to the introduction of Se, which lowers the phonon energy in the SrZnOSe lattice, thereby reducing emission loss due to non‐radiative relaxation during ML process. Furthermore, the application of AZnOS in vivo faces significant challenges. Due to their large size and irregular shapes, unprocessed AZnOS particles are difficult to inject into living organisms. Compared to nanoparticles such as NaY(Gd)F_4_: Ln^3+^ and ZnGa_2_O_4_: Cr^3+^,^[^ ^193^, ^194^ ^]^ AZnOS struggles to achieve effective transport within vascular tissues. Therefore, further enhancement and modification studies are required to enable more intensive applications of AZnOS within the NIR range.

It is well known that green plants convert light energy into chemical energy through photosynthesis, which provides the base energy for ecosystems and supports the means of production needed for human activities. However, there is a significant mismatch between the absorption spectra of plant photosynthetic pigments, like *chlorophylls (a,b*), and the solar spectrum: most plants only absorb blue (400–500 nm) and red (600–700 nm) light with high efficiency, while green and some yellow light bands cannot be effectively utilized, resulting in the waste of this part of solar energy.^[^ ^135^, ^136^, ^146^ ^]^ Therefore, by converting solar light bands that cannot be effectively utilized by plants to red or blue light, the efficiency of plant photosynthesis can be further promoted by increasing the utilization rate of solar light, which can then optimize the growth of organs such as fruits, roots, stems, and leaves. As a proof‐of‐concept, Lian et al. innovatively prepared CaS: Eu^2+^/Pb^2+^@CaZnOS: Pb^2+^ with a core–shell structure and added them to plastic films or laminated glass used in greenhouses.^[^ ^136^ ^]^ As shown in Figure 20c, the excitation spectrum of this sample was highly matched with the solar spectrum, while its emission spectrum precisely overlapped with the absorption spectra of *chlorophylls (a,b)*, thus significantly enhancing the photosynthetic efficiency of greenhouse crops. This study provides a new strategy for the development of AZnOS‐based phosphors for photo‐ecological agricultural applications and expands the practical path of inorganic luminescent materials in agriculture.

## 6 Conclusions and Perspectives

This review systematically examines the crystal structure and intrinsic optical properties of AZnOS. By leveraging their exceptional structural stability as luminescent matrices, we analyze their photophysical behavior upon doping with single or multiple luminescent ions. Key properties discussed include PL, ML, and other forms of luminescence. Additionally, we explore doping control strategies, such as multimode excitation modulation, alongside preparation methodologies and the application potential of these functional materials.

### 6.1 Advancements and Strategic Role

Driven by advancements in atomic‐scale simulations, quantum chemical modeling, and crystallographic analysis, AZnOS‐based phosphors have undergone transformative developments in luminescence technology, establishing themselves as cornerstone materials in next‐generation solid‐state lighting. Their exceptional performance stems from a unique combination of structural rigidity and electronic flexibility, enabling precise control over energy transfer pathways when doped with luminescent ions. For instance, Ln^3+^‐doped AZnOS benefits from efficient *f*–*f* transitions and minimized non‐radiative decay, while TM‐doped variants leverage *d*–*d* orbital splitting modulated by crystal field effects, resulting in quantum yields exceeding 90% under optimized conditions. The tunability of their spectral profiles spanning UV to NIR regions is particularly advantageous for applications requiring tailored emission wavelengths, such as white LEDs with high color rendering indices (>90) or plant growth lighting.^[^ ^18^, ^136^, ^195^ ^]^ Notably, Mn^2+^‐doped AZnOS exhibit a sharp red emission peak at ≈620 nm, addressing the “green gap” challenge in *pc*‐LEDs. Meanwhile, ML properties of AZnOS, originating from piezoelectric‐induced charge redistribution, enable stress‐responsive sensing layers with sub‐millisecond response times, as demonstrated in smart touchscreens and structural health monitoring systems. The ML phenomenon in AZnOS has inspired innovations like US‐triggered drug release systems and in vivo temperature probes, highlighting its biomedical potential.^[^ ^78^, ^164^, ^165^ ^]^ Furthermore, the maturity of this research is demonstrated through systematic doping strategies, such as achieving charge balance between dopant ions (like Ln^3+^) and A^2+^ in AZnOS via co‐doping with charge compensators. Despite these achievements, critical gaps remain, including thermal stability under high‐power *pc*‐LEDs operation and scalable synthesis of pure‐phase AZnOS.

Thus, this review synthesizes recent breakthroughs, contextualizes them within the broader framework of inorganic luminescent materials, and outlines emerging frontiers. By dissecting structure–property relationships and benchmarking against commercial standards, we aim to catalyze interdisciplinary research at the nexus of materials science, photonics, and quantum engineering. Future development directions may prioritize hybrid integration with QDs to generate tunable luminescence or defect engineering, thereby unlocking single‐photon emission capabilities and further solidifying AZnOS's role in the field of photonic.

### 6.2 Challenges and Future Directions

Despite significant strides in unraveling optical phenomena and broadening application scopes, the luminescent materials field remains constrained by structural and compositional limitations when compared to legacy platforms. Traditional luminescent systems, such as RE‐doped oxides (e.g., YAG: Eu^2+^), while stable, often suffer from high production costs, environmental toxicity, and limited spectral tunability.^[^ ^11^ ^]^ The AZnOS‐based phosphors, though promising, face synthesis challenges: conventional solid‐state reactions require harsh temperatures (>1000 °C) and prolonged annealing, yielding micron‐sized particles with low surface areas that hinder device integration.^[^ ^188^ ^]^ Even high‐performance materials, such as QDs, grapple with stability issues under ambient conditions or scalability bottlenecks in ligand‐free synthesis.^[^ ^196^ ^]^

Therefore, innovative preparation processes are critical to ensuring economic and eco‐friendly production. Future breakthroughs may focus on hybrid synthesis strategies. For example, combining sol–gel processing with microwave‐assisted crystallization not only reduces the synthesis temperature to below 500 °C, but also enables rapid, phase‐controlled synthesis of AZnOS‐based phosphors.^[^ ^148^, ^153^, ^162^, ^183^ ^]^ Moreover, the luminescence performance of phosphors can be significantly enhanced by constructing ordered defect structures in the host lattice. The current process technology has been able to realize the orderly introduction and precise regulation of specified defects (e.g., oxygen vacancies) in the lattice, which opens up a new path for defect engineering to improve the material properties.^[^ ^196^ ^]^ Notably, since the 2023 Nobel Prize in Chemistry was awarded to QDs research, the field has become a revolutionary frontier in nanotechnology.^[^ ^197^ ^]^ Based on the unique optical properties exhibited by quantum confinement effects, QDs technology is leading us towards the development of smaller‐sized phosphors. In order to fully utilize the performance advantages of these new materials, it is necessary to optimize the synthesis process and doping strategy simultaneously: through fine‐tuning of synthesis parameters and efficient doping design, higher quantum yield and wider application scenarios can be achieved simultaneously. In addition, the rapid development of Artificial Intelligence (AI) has provided a new means of accelerating the discovery of high‐performance phosphors. AI models trained based on high‐throughput combinatorial libraries are able to predict optimal doping concentrations or reaction paths, thereby significantly reducing the time‐consuming and costly traditional trial‐and‐error experiments.^[^ ^198^ ^]^ From hybrid synthesis to defect engineering, from QDs innovation to AI‐enabled, the synergistic innovation of multiple technology dimensions is driving the phosphor preparation process to evolve in the direction of more efficient, controllable, and environmentally friendly.

In summary, while luminescent materials have made remarkable progress, their full potential remains hindered by synthesis‐related constraints. By prioritizing eco‐design, scalability, and interdisciplinary innovation, the field can transition from incremental improvements to transformative advancements, enabling ubiquitous integration into energy‐efficient lighting, displays, and beyond.

### 6.3 Vision for Breakthrough Applications

Looking ahead, next‐generation luminescent phosphors, engineered through strategic design principles, are poised to revolutionize sustainable energy technologies while mitigating environmental impacts across their lifecycle. In solid‐state lighting, for instance, AZnOS‐based red phosphors with quantum yields surpassing 50% could reduce energy consumption at least by 20% compared to conventional Eu^2+^‐doped oxides, directly lowering carbon emissions from power generation.^[^ ^109^, ^130^ ^]^ Beyond lighting, these materials hold promise for enhancing photovoltaic cell efficiencies via spectral conversion of sub‐bandgap photons, or as photocatalysts for water splitting, where their tunable bandgaps could optimize solar fuel production yields.^[^ ^135^, ^136^ ^]^ The environmental dividends extend beyond energy savings. By replacing toxic activators like Pb^2+^ with earth‐abundant TM (e.g., Mn^2+^ and Cu^+^) in AZnOS, the field can mitigate ecological risks associated with mining and disposal.^[^ ^114^, ^115^, ^121^ ^]^ Furthermore, innovative synthesis protocols, such as microwave‐assisted reaction, could reduce process energy demands by more than 50% compared to traditional solid‐state methods, aligning with circular economy principles.^[^ ^148^, ^153^, ^154^ ^]^

To unlock these possibilities, this review underscores the imperative for dual innovation: material design and process engineering. On the material front, hybrid AZnOS nanostructures, combining quantum confinement effects with defect engineering, could enable high‐performance tunable emission of nanoparticles and simplify *pc*‐LEDs device architectures.^[^ ^160^, ^196^ ^]^ At the same time, breakthroughs in mechanochemical synthesis, such as cryomilling at liquid N_2_ temperatures, offer pathways to metastable polymorphs with enhanced ML responses, critical for stress‐sensing applications in aerospace or biomedical devices. Interdisciplinary synergies will be key; for example, AI models can be used to predict optimal doping configurations.^[^ ^198^ ^]^ It is also important to consider how to industrialize the product and successfully translate laboratory‐scale trials into scalable manufacturing processes. Ultimately, the vision extends beyond incremental gains. By integrating AZnOS‐based phosphors with emerging technologies, the field can pioneer entirely new paradigms. Such innovations demand not only scientific creativity but also policy frameworks that incentivize eco‐conscious material development, ensuring a sustainable and equitable transition to advanced photonic technologies.

## Conflict of Interest

The authors declare no conflict of interest.
